# Psychometric properties of the Persian version of the chemotherapy side effects fear scale among Iranian patients with cancer

**DOI:** 10.1371/journal.pone.0354374

**Published:** 2026-08-13

**Authors:** Mohammad Eghbali, Hossein Bakhtiari-Dovvombaygi, Seyyed Hamid Hoseini, Razieh Bandari, Amir Hossein Dehghan, Mohammadreza Askari, Adeleh Rezagholizadeh Shirvan, Reza Negarandeh

**Affiliations:** 1 Department of Nursing, School of Nursing and Midwifery, Torbat Heydariyeh University of Medical Sciences, Torbat Heydariyeh, Iran; 2 Health Sciences Research Center, Torbat Heydariyeh University of Medical Sciences, Torbat Heydariyeh, Iran; 3 Nursing & Midwifery Care Research Center, School of Nursing and Midwifery, Tehran University of Medical Sciences, Tehran, Iran; 4 Student Research Committee, Tabriz University of Medical Sciences, Tabriz, Iran; 5 Department of Cardiology, 9 Day Educational Hospital, Torbat Heydariyeh University of Medical Sciences, Torbat Heydariyeh, Iran; 6 Department of Community Medicine, School of Medicine, Semnan University of Medical Sciences, Semnan, Iran; 7 Department of Surgical and Anesthesia Technology, School of Allied Medical Sciences, Semnan University of Medical Sciences, Semnan, Iran; 8 Nursing and Midwifery School, Student Research Committee, Tehran University of Medical Sciences, Tehran, Iran; 9 Student Research Committee, School of Nursing and Midwifery, Tehran University of Medical Sciences, Tehran, Iran; 10 Department of Nursing, School of Nursing and Midwifery, Torbat Heydariyeh University of Medical Sciences, Torbat Heydariyeh, Iran; 11 Department of Palliative Care, Cancer Institute, Imam Khomeini Hospital, Tehran University of Medical Sciences, Tehran, Iran; Tarbiat Modares University Faculty of Medical Sciences, IRAN, ISLAMIC REPUBLIC OF

## Abstract

Fear of chemotherapy-induced side effects is a major concern among patients with cancer and may affect psychological well-being and treatment adherence. This methodological study aimed to translate, culturally adapt, and evaluate the psychometric properties of the Persian version of the Chemotherapy Side Effects Fear Scale (CheSeFS). The study was conducted from January to April 2024 at the Cancer Institute of Imam Khomeini Hospital Complex in Tehran, Iran, and included 600 patients undergoing chemotherapy. The CheSeFS underwent forward–backward translation and cross-cultural adaptation. Face and content validity, structural validity, convergent and discriminant validity, internal consistency, test–retest reliability, measurement error, a distribution-based MIC estimate, and factors associated with CheSeFS scores were evaluated. All statistical analyses were performed using Jamovi version 2.7.1 and included descriptive statistics, item analysis, exploratory factor analysis, confirmatory factor analysis, reliability and measurement error analyses, convergent and discriminant validity assessment, distribution-based MIC estimation, and regression analysis. Patient and expert evaluations supported face and content validity. Exploratory factor analysis identified a two-factor structure, including Physiological fear and Subjective or Psychological fear, explaining 54.4% of the total variance. Three items were removed during factor analysis, resulting in a final 11-item scale. Confirmatory factor analysis supported model fit, with χ²(df = 43) = 84.2, RMSEA = 0.06, CFI = 0.99, TLI = 0.99, and SRMR = 0.04. Internal consistency was satisfactory, with Cronbach’s alpha = 0.89 for the total scale. Measurement error analysis showed an SEM of 1.64 and an MDC95 of 4.56, and the distribution-based MIC estimate was 1.01 points. The Persian 11-item CheSeFS demonstrated satisfactory validity and reliability for assessing chemotherapy-related fear among Iranian patients with cancer.

## 1. Introduction

Cancer has emerged as a major public health concern, ranking as the second leading cause of mortality worldwide. According to the American Cancer Society’s (Global Cancer Statistics 2024) report, approximately 20 million new cancer cases were diagnosed worldwide in 2022, and about 9.7 million individuals died from the disease [1–3]. The global cancer burden is projected to continue rising, reaching an estimated 28.4 million new cases by 2040 [4]. This increasing prevalence of cancer imposes significant physical, emotional, and financial strain on individuals, families, communities, and healthcare systems worldwide [5].

Reflecting the global increase in cancer burden, Iran continues to face a substantial cancer burden. According to the latest GLOBOCAN report, published in 2024 and presenting estimates for 2022, more than 137,000 new cancer cases and approximately 87,000 cancer-related deaths were estimated in Iran [6].

The distribution of cancer types varies considerably across populations and geographical regions, reflecting differences in demographic structure, socioeconomic conditions, lifestyle-related exposures, environmental and occupational risk factors, and access to prevention and early detection services [1]. Among men, lung and prostate cancers are the most commonly diagnosed cancers worldwide, whereas among women, breast cancer is the most common, followed by lung and colorectal cancers [7]. In Iran, the most commonly diagnosed cancers in 2022 were stomach, breast, colorectal, lung, and prostate cancers, which together accounted for nearly half of all new cancer cases. Among Iranian men, stomach, prostate, and lung cancers were the leading cancers, whereas among women, breast cancer was the most common, followed by stomach and colorectal [8]. Globally, cancer remains one of the leading causes of morbidity and mortality in both high-income and low- and middle-income countries [1]. Cancer management encompasses a range of therapeutic approaches, with surgery, radiation therapy, and chemotherapy serving as the primary modalities [9]. Surgical interventions and radiation therapy are localized treatments, typically planned and completed within a few weeks, and usually result in mainly localized side effects. In contrast, chemotherapy, a systemic treatment commonly employed for various cancer types, is highly effective in improving survival rates but may be administered over months or even years, depending on the specific malignancy. Due to its systemic nature, chemotherapy often causes a range of side effects, some of which can be life-threatening [10,11]. The spectrum of chemotherapy-induced side effects varies significantly based on the characteristics of the drug administered, including its dosage, form, route of administration, and interactions with other medications [12].

In recent years, patients’ perceptions of chemotherapy-related side effects have shifted, placing greater emphasis on psychological impacts such as emotional, social, and sexual functioning, rather than solely physical manifestations like hair loss, fatigue, and anemia-induced weakness [13]. Alongside these changes, many patients experience various forms of fear during treatment, including anxiety, depression, and the fear of relapse.

Fear is conceptualized as an emotional response to a specific and identifiable threat, whereas anxiety represents a more diffuse and future-oriented state of apprehension [14]. In the context of chemotherapy, fear of side effects represents a treatment-specific construct directly linked to identifiable physical and social consequences of therapy [15]. Therefore, general psychological distress measures may not adequately capture the nuanced, situational, and treatment-specific nature of fears related to chemotherapy.

Moreover, fear related to chemotherapy side effects has been associated with treatment delay, reduced adherence [16], anticipatory nausea [17], and diminished quality of life [18]. Given the clinical importance of this construct, an essential scale is culturally adapted and psychometrically validated.

Although several scales, such as the Beck Anxiety Inventory [19], Hamilton Anxiety Rating Scale (HAM-A), Zung Self-Rating Anxiety Scale [10], Depression Anxiety Stress Scales (DASS) [20], and Hospital Anxiety and Depression Scale (HADS ([21], assess general psychological distress (e.g., anxiety and depression), they are not specifically designed to capture fear related to chemotherapy side effects. These scales primarily evaluate broad emotional states and are not sensitive to treatment-specific concerns such as anticipatory nausea, fear of alopecia, or treatment-induced neuropathy. Moreover, other scales like the Fear of Cancer Recurrence Inventory (FCRI) focus on the fear of disease recurrence rather than treatment-related adverse effects [22]. To address this need, the Chemotherapy Side Effects Fear Scale (CheSeFS) offers a promising solution. This scale was originally developed and psychometrically validated by its Serbian creators [23] to assess fears related to chemotherapy side effects. However, further evaluation of its psychometric properties in translated versions, including cultural adaptations, is necessary to ensure its reliability and validity across different populations.

The current research aims to adapt the CheSeFS to Persian, focusing on its cultural relevance and psychometric evaluation within the Iranian context. This study seeks to validate the Persian version of the CheSeFS, providing a reliable scale for assessing the fear of chemotherapy-related side effects among Iranian patients with cancer. The ultimate goal is to establish its applicability for future research and clinical practice in Iran.

## 2. Materials and methods

### 2.1. Study design and settings

This methodological study took place between January 2024 and April 2024. The study was conducted in clinics of the Cancer Institute at Imam Khomeini Hospital Complex, affiliated with the Tehran University of Medical Sciences. Psychometric properties of the CheSeFS, including face and content validity, construct validity (exploratory factor analysis (EFA) and confirmatory factor analysis (CFA)), and reliability (stability and internal consistency) were evaluated. The selection and reporting of measurement properties were guided by relevant COSMIN recommendations. Responsiveness and criterion validity were not evaluated because this cross-sectional methodological study did not include longitudinal change data, an external anchor, or an appropriate gold standard [24].

### 2.2. Sample size, inclusion, and exclusion criteria

The sample size was determined to be 300 according to the Comrey and Lee guidelines [24]. Participants were chosen through a convenience sampling from the Cancer Institute Hospital of Imam Khomeini University in Tehran. To reduce bias and improve the robustness of the factor structure, the total sample of 600 participants was divided into two independent, non-overlapping groups. EFA was conducted on the first group (n = 300), and CFA was performed on the second group (n = 300). The inclusion criteria were as follows: individuals (1) aged 18 years or older, (2) diagnosed with cancer and undergoing chemotherapy, (3) without comprehension and learning difficulties, and (4) provided informed consent to participate in the study. Recruitment continued until the desired sample size was reached. The exclusion criteria included a lack of willingness to continue participation and filling in the questionnaire incompletely.

### 2.3. Measurements

Data were collected using the Demographic and Clinical Information form and the chemotherapy side effects Fear Scale (CheSeFS). The de-identified dataset used for the EFA, CFA, and ICC calculation is provided as S1, S2, and S3 Data respectively.

#### 2.3.1. Demographic and clinical information form.

This researcher-developed form includes variables related to both demographic and clinical characteristics. Demographic variables include gender, marital status, age group, education level, employment status, smoking status, and region of residence. Clinical characteristics cover the method of chemotherapy drug administration, the type of cancer, family history of cancer, the presence of pain, and chronic diseases.

#### 2.3.2. Chemotherapy side effects fear scale.

This scale was developed by Ivana Vasić and colleagues in Serbia in 2017. It consists of 14 items measured on a 5-point Likert scale. The scale assesses fears related to loss of appetite, hair loss, inability to spend time with friends, experiencing pain during treatment, rapid weight loss, use of antidepressants, the occurrence of mouth sores, anemia, nausea and fatigue, persistent diarrhea, sleep disturbances, and others. EFA identified two factors: Physiological fear and Subjective fear, explaining 61% of the variance. Furthermore, the questionnaire demonstrated excellent reliability with a Cronbach’s alpha of 0.922, confirming its strong internal consistency [15].

### 2.4. Translation of the CheSeFS

After receiving approval from Dr. Ivana Vasić, the developer of the scale, the scale was then translated according to the translation and cross-cultural adaptation guideline from Beaton et al. [25]. Accordingly, the English version of the CheSeFS was initially translated into Persian using a forward-backward method, which included the following steps: (1) In the forward translation stage, two independent translations were conducted by bilingual translators whose mother tongue was Persian. One translator was familiar with the clinical concepts, while the other was naïve to the constructs, ensuring both conceptual accuracy and linguistic naturalness (2). Subsequently, the two forward translations (T1 and T2) were compared with the original version and synthesized into a single reconciled Persian version (T-12). Discrepancies were resolved through consensus and documented afterward. (3) In the back-translation stage, two independent translators whose mother tongue was English and who were blinded to the original version performed back-translations (BT1 and BT2) of the reconciled Persian version (T-12). These translators had no involvement in the initial phase and were not informed about the study concepts. (4) In the expert committee stage, including two psychometricians, two nursing faculty members, an oncologist, and the translators (forward and back translators), all available versions of the scale (original, T1, T2, T-12, BT1, and BT2) were examined. Semantic, idiomatic, experiential, and conceptual equivalences were evaluated, and discrepancies were resolved through consensus, ultimately resulting in a pre-final unified Persian version. During the expert committee review and cross-cultural adaptation process, the wording of the items was evaluated for semantic, idiomatic, experiential, and conceptual equivalence. The committee specifically considered whether the translated terms were understandable for Iranian patients undergoing chemotherapy, culturally appropriate, and clinically equivalent to the original items. Minor wording revisions were made to improve the clarity and naturalness of Persian phrasing. No item was added, removed, or substantially changed during the translation, expert committee review, or cross-cultural adaptation stages. The pre-final Persian version was then used for face validity and cognitive debriefing with patients.

### 2.5. Face validity

In this study, the face validity of the scale was assessed through a combination of qualitative and quantitative methods.

Qualitative face validity was evaluated through cognitive interviews with 10 patients undergoing chemotherapy. The pre-final Persian version of the CheSeFS was provided to the patients, and they were asked to evaluate the clarity, comprehensibility, relevance, level of difficulty, cultural appropriateness, and ease of response for each item. Patients were also asked whether any words, phrases, or items were unclear, confusing, sensitive, or culturally inappropriate.^32^. Their comments were reviewed by the research team, and minor wording revisions were made where necessary to improve clarity and naturalness of Persian phrasing. No item was added or removed during the face validity assessment.

For quantitative face validity, the same 10 patients rated the importance and suitability of each item using a 5-point Likert scale. A score of 5 indicated complete importance/suitability, whereas a score of 1 indicated complete lack of importance/suitability. The item impact score was then calculated using the following formula: Impact Score = Frequency × Importance/Suitability [26]. Items with impact scores greater than 1.5 were considered acceptable and were retained for further psychometric evaluation.

### 2.6. Content validity

Content validity was assessed using both qualitative and quantitative approaches to evaluate whether the Persian version of the CheSeFS adequately represented the intended construct and was appropriate for use among Iranian patients undergoing chemotherapy.

In the qualitative phase, the pre-final Persian version was reviewed by 10 experts with expertise in psychometric assessment (n = 4), nursing (n = 3), and oncology (n = 3). The experts evaluated the items in terms of relevance, clarity, simplicity, wording, grammatical accuracy, cultural appropriateness, item placement, and the clarity of the scoring instructions. Their comments were reviewed by the research team, and any required revisions were made through consensus. This process was used to ensure that the items were conceptually equivalent to the original version and culturally appropriate for the Iranian context.

In the quantitative phase, the content validity ratio (CVR) and item-level content validity index (I-CVI) were calculated. For CVR, the experts rated the necessity of each item using a three-point scale: essential, useful but not essential, and not necessary. The CVR for each item was calculated using Lawshe’s formula: CVR = (ne − N/2)/ (N/2), where represents the number of experts rating the item as essential and N represents the total number of experts. Based on Lawshe’s critical values, for a panel of 10 experts, a minimum CVR value of 0.62 was considered acceptable [27]. For I-CVI, the experts rated each item in terms of relevance, clarity, and simplicity using a four-point scale: 1 = not relevant/not clear/not simple, 2 = needs major revision, 3 = relevant/clear/simple but needs minor revision, and 4 = highly relevant/clear/simple. The I-CVI was calculated as the proportion of experts rating each item as 3 or 4. An I-CVI value above 0.79 was considered acceptable, values between 0.70 and 0.79 indicated the need for revision, and values below 0.70 were considered unacceptable [28]. Items meeting the accepted CVR and I-CVI thresholds were retained for further psychometric evaluation.

### 2.7. Preliminary item analysis

Before conducting exploratory factor analysis, item performance was evaluated using corrected item–total correlations, internal consistency indices, descriptive statistics, missing-response patterns, floor and ceiling effects, and response ease. Corrected item–total correlations below 0.30 were considered weak and were considered for removal. Descriptive statistics, including mean, standard deviation, skewness, and kurtosis, were calculated to examine score distribution characteristics.

Floor and ceiling effects were assessed by calculating the percentage of participants who obtained the minimum and maximum possible scores for each subscale and the total score. Floor or ceiling effects were considered present if more than 15% of participants obtained the lowest or highest possible score. Response ease was evaluated using patient feedback during cognitive debriefing and by examining missing or incomplete responses in the field-test dataset.

Measurement error was assessed using the standard error of measurement (SEM) and the minimal detectable change at the 95% confidence level (MDC95), based on the test–retest reliability data. SEM was calculated as SD × √ (1 − ICC), where SD represents the standard deviation of the total score at the first administration and ICC represents the single-measure intraclass correlation coefficient. MDC95 was calculated as 1.96 × √2 × SEM. Because this methodological study did not include a longitudinal external anchor or patient-rated global change criterion, an anchor-based Minimal Important Change (MIC) was not estimated. However, as supportive distribution-based evidence for score interpretability, a distribution-based MIC estimate was calculated using the small effect size criterion: MIC = 0.20 × SD.

### 2.8. Construct validity

#### 2.8.1. Exploratory Factor Analysis (EFA).

In the first step of the construct validity of the scale, EFA was performed on a sample of 300 participants using Jamovi software (version 2.7.1) to extract the number of latent variables. The Kaiser-Meyer-Olkin (KMO) and Bartlett’s tests were used to assess the suitability of the data and the adequacy of the sample size. A KMO score between 0.7 and 0.8 was regarded as acceptable, while a score from 0.8 to 0.9 was considered excellent. A value of P < 0.05 is considered significant in Bartlett’s test.

Factor extraction was performed using the maximum likelihood method, followed by Promax oblique rotation to allow for correlated factors. The number of factors to retain was determined using parallel analysis in conjunction with inspection of the scree plot, which provided a more robust estimate of the appropriate number of latent constructs. Items with factor loadings below 0.40 or those lacking conceptual consistency with the extracted factors were considered for removal to achieve a stable and interpretable factor structure [28]

#### 2.8.2. Confirmatory Factor Analysis (CFA).

To validate the factor structure identified in the EFA, CFA was conducted on a separate sample of 300 participants using Jamovi software (version 2.7.1) with the SEM (lavaan-based) module. Before CFA, the adequacy of the CFA sample was evaluated based on the number of participants, the number of retained items, and the stability of model estimation. Considering the final 11-item structure, the CFA sample provided more than 25 participants per retained item, exceeding commonly recommended participant-to-item ratios for factor analysis. The CFA model was also examined for convergence, standardized factor loadings, residuals, and model fit indices.

The CFA aimed to confirm the factor structure identified in the EFA, with items assigned to each factor as determined by the previous analysis. Model fit was evaluated using several indices, including the Comparative Fit Index (CFI), Tucker-Lewis Index (TLI), Standardized Root Mean Square Residual (SRMR), Root Mean Square Error of Approximation (RMSEA), and χ² (Chi-square) with degrees of freedom [29]. The thresholds for acceptable model fit were set at > 0.9 for CFI, > 0.9 for TLI, < 0.08 for SRMR, < 0.08 for RMSEA, and a non-significant χ² value (p > 0.05) with appropriate [30,31].

To assess predictors of total scores on the CheSeFS, a linear regression analysis was conducted. Prior to performing the analysis, several assumptions were tested to ensure the validity of the model. The normality of residuals was evaluated using the Kolmogorov-Smirnov test (p = 0.12) and visualized with Q-Q plots, which indicated that the residuals largely followed a normal distribution with minimal deviations in the tails. Multicollinearity was assessed using the Variance Inflation Factor (VIF), and all predictors demonstrated VIF values below the threshold of 10, confirming no severe collinearity issues. Residual independence was tested using the Durbin-Watson statistic, which showed no significant autocorrelation. These steps ensured that the assumptions of linear regression were met, allowing for reliable interpretation of the results.

#### 2.8.3. Convergent and discriminant validity.

Convergent and discriminant validity were assessed following confirmatory factor analysis using Jamovi software (version 2.7.1) with the SEM (lavaan-based) module. Convergent validity was evaluated using Composite Reliability [31] and Average Variance Extracted (AVE), which respectively reflect the internal consistency of items within a construct and the amount of variance captured by the latent factor. CR values above 0.70 and AVE values greater than 0.50 were considered indicative of adequate convergent validity [31].

Discriminant validity was examined using the Heterotrait–Monotrait ratio (HTMT), which assesses the distinctiveness of latent constructs. HTMT values below 0.85 were considered indicative of satisfactory discriminant validity [32].

### 2.9. Reliability

To assess reliability, both Cronbach’s alpha and McDonald’s ω were calculated to evaluate internal consistency for the total score as well as the sub-scores. For adequate internal consistency, the Cronbach’s alpha should be 0.7 or higher [33]. Additionally, the test-retest method was used over a two-week period with a sample of 30 participants from the target population. Scores from the two testing sessions were compared to measure the consistency of the test-retest results, and the Intraclass Correlation Coefficient (ICC) was computed to determine test-retest reliability. An ICC index greater than 0.80 is considered to indicate a desirable level of stability [31].

### 2.10. Ethical considerations

The study received approval from the Research Ethics Committee at Tehran University of Medical Sciences (Ethical code: IR.TUMS.MEDICINE.REC.1401.156). Necessary permissions were obtained from the relevant authorities at the research sites and the developer of the original scale. Patients were thoroughly informed about the study’s purpose, their right to withdraw at any time, and the confidentiality of their information. Written informed consent was obtained from all participants. All procedures were conducted in full compliance with the applicable guidelines and regulations.

## 3. Results

### 3.1. Characteristics of the participants

This study included 600 cancer patients with cancer undergoing chemotherapy, who visited the Cancer Institute of Imam Khomeini Hospital in Tehran between January 2024 and April 2024. The mean age of the participants was 47.73)10.84(years. Among these patients, 74.0% were women. The majority of the sample had a high school diploma (45.3%), were employed (55.7%), and were single (46.7%). More than one-third of the patients were diagnosed with gastrointestinal cancer (42.0%), and the majority received chemotherapy through peripheral veins (73.7%). Additionally, over half of the patients had a family history of cancer (60.7%), and 45.0% were smokers. Details are provided in Table 1.

**Table 1:** Socio-demographic and Clinical status characteristics of the participants in (N = 600).

Variables	Category	Mean (SD) or n (%)
**Age (years)**	–	47.73 (10.84)
**Pain score (0–10)**	–	1.37 (1.72)
**Gender**	Male	156 (26.0)
	Female	444 (74.0)
**Marital status**	Single	280 (46.7)
	Married	256 (42.7)
	Divorced/separated	64 (10.7)
**Type of venous access**	Central	158 (26.3)
	Peripheral	442 (73.7)
**Education level**	Illiterate	38 (6.3)
	Diploma	272 (45.3)
	Associate degree	56 (9.3)
	Bachelor’s degree	188 (31.3)
	Master’s or higher degree	46 (7.7)
**Employment status**	Unemployed	266 (44.3)
	Employee	334 (55.7)
**Type of cancer**	Breast	192 (32.0)
	Gastrointestinal	252 (42.0)
	Skin	30 (5.0)
	Lung	88 (14.7)
	Brain and spinal cord	38 (6.3)
**Cancer in Family**	Yes	364 (60.7)
	No	236 (39.3)
**Region**	City	480 (80.0)
	village	120 (20.0)
**Chronic Disease**	Yes	150 (25.0)
	No	450 (75.0)
**Smoking**	Yes	270 (45.0)
	No	330 (55.0)

### 3.2. Face and content validity

During qualitative face validity and cognitive debriefing, participants reported that the pre-final Persian version of the CheSeFS was clear, understandable, relevant, culturally appropriate, and easy to respond to. Patient comments led only to minor wording revisions to improve clarity and naturalness of Persian phrasing. No item was added or removed during the face validity assessment. In the quantitative face validity assessment, the impact score for every item exceeded 1.5, indicating that all items were appropriate and were retained for further psychometric evaluation.

Content validity was confirmed based on the feedback of 10 experts in psychometrics, nursing, and oncology. The experts confirmed the relevance, clarity, simplicity, cultural appropriateness, item placement, and scoring instructions of the Persian version. The CVR for all items in the CheSeFS was equal to 1.00, indicating that no items were excluded based on the CVR criterion. Furthermore, the I-CVI values ranged from 0.80 to 1.00, with all items meeting the minimum acceptable threshold. No new items were suggested by patients or experts during the face and content validity assessments. These findings indicated that the translated items were conceptually equivalent to the original version, culturally relevant, and comprehensive for the Iranian context.

### 3.3. Preliminary item analysis and measurement error

Preliminary item analysis was conducted before exploratory factor analysis. Corrected item–total correlations ranged from 0.383 to 0.783, and all items exceeded the minimum acceptable criterion of 0.30. This indicated satisfactory item homogeneity before factor extraction.

Descriptive statistics for the final CheSeFS items are presented in Table 2. The mean item scores ranged from 1.56 to 1.92. Skewness and kurtosis values were within acceptable ranges, suggesting no substantial deviation from normal distribution and indicating appropriate variability of responses across items.

**Table 2:** Descriptive statistics of CheSeFS items by factor (N = 600).

Factor	Item	Mean	SD	Skewness	Kurtosis
**Factor1: Physiological fear**	**Q1**	1.66	0.68	0.60	−0.46
	**Q2**	1.76	0.83	0.54	−1.12
	**Q5**	1.76	0.73	0.41	−1.05
	**Q7**	1.92	0.88	0.46	−0.89
	**Q8**	1.72	0.75	0.51	−1.07
	**Q9**	1.81	0.79	0.75	0.10
	**Total**	10.6	3.84	0.63	−0.75
**Factor2: Subjective or Psychological** **fear**	**Q3**	1.74	0.68	0.37	−0.83
	**Q10**	1.63	0.57	0.23	−0.74
	**Q11**	1.79	0.64	0.22	−0.66
	**Q12**	1.76	0.59	0.12	−0.48
	**Q13**	1.56	0.50	−0.16	−1.78
	**Total**	8.48	2.22	0.15	−0.94

Frequency analyses included all 600 participants, indicating no missing responses in the variables included in these analyses. In addition, all 30 participants included in the test–retest analysis had valid data, and no cases were excluded. These findings supported good response ease and completeness of responses.

No substantial floor or ceiling effects were observed. For the total score, 4.0% of participants obtained the minimum possible score, and no participant obtained the maximum possible score. For the Physiological fear subscale, 11.7% of participants obtained the minimum possible score, and no participant obtained the maximum possible score. For the Subjective or Psychological fear subscale, 10.7% of participants obtained the minimum possible score, and no participant obtained the maximum possible score. Therefore, all floor and ceiling values were below the predefined 15% threshold.

Measurement error was assessed using the test–retest data. The standard deviation of the total score at the first administration was 5.03, and the single-measure ICC was 0.893. The SEM for the total score was 1.64, and the MDC95 was 4.56. Using the small effect size criterion, the distribution-based MIC estimate for the total CheSeFS score was 1.01 points (0.20 × 5.03). This estimate was lower than the MDC95, suggesting that although a 1-point change may represent a small distribution-based change, it may not exceed measurement error at the individual level.

### 3.4. Construct validity

#### 3.4.1. Exploratory factor analysis.

**Fig 1:**
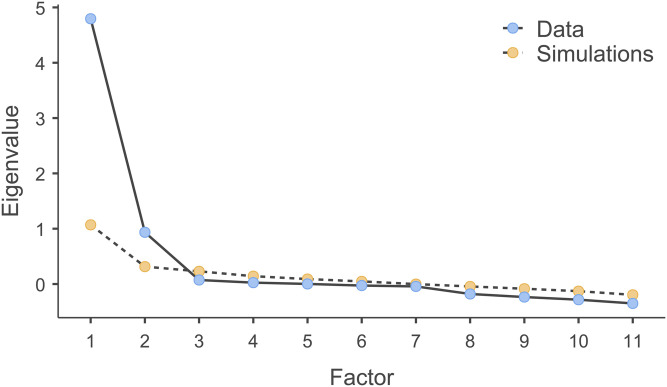
Scree plot of exploratory factor analysis for the Persian version of CheSeFS (N = 300).

The KMO Measure of Sampling Adequacy for this factor analysis model was 0.91, indicating that the sample size was sufficient for the analysis. Additionally, Bartlett’s test of sphericity was significant (χ² = 3240, df = 55, p < 0.001), confirming the appropriateness of the data for factor analysis. Parallel analysis supported the retention of a two-factor solution, which was further supported by inspection of the scree plot (Fig 1). The final two-factor model explained 54.4% of the total variance, with the first factor accounting for 34.1% and the second for 20.4%

During the iterative maximum likelihood extraction with Promax rotation, three items were removed to achieve a stable and interpretable structure.

Item 4 (“I am afraid of severe pain during the therapy”) demonstrated relatively high uniqueness and weak loading, indicating limited shared variance with the underlying constructs. Conceptually, pain during therapy may reflect an acute procedural concern rather than sustained fear of chemotherapy side effects, which may explain its instability within the factor structure.

After removal of Item 4, Item 6 (“I am afraid of having to take antidepressant drugs”) loaded onto a separate third factor as a single-item construct. Single-item factors are generally considered unstable. Conceptually, this item may capture broader psychological concerns and subsequent treatment needs, which did not align closely with the remaining side-effect fear items in the extracted structure; therefore, it was excluded.

Subsequently, Item 14 (“I am afraid of lasting side effects on my heart, lung, kidneys, etc.”) formed a single-item third factor despite acceptable loading values. Although statistically adequate, this item reflects long-term systemic complications that differ conceptually from the immediate physiological and subjective experiences represented in the retained factors. Its removal resulted in a clearer and theoretically coherent two-factor structure.

The final model retained Items 1, 2, 5, 7, 8, and 9 under Factor 1 (Physiological fear component), and Items 3, 10, 11, 12, and 13 under Factor 2 (Subjective or Psychological fear component). Factor loadings ranged from 0.53 to 0.85 (Table 3).

**Table 3:** The Maximum likelihood extraction method was used in combination with a ‘Promax’ rotation items of the scale (N = 300).

Factor (Subscale)	Item	Factor Loadings	Variance Explained (%)
**Factor1: Physiological fear**	Q1	0.75	34.1%
	Q2	0.75	
	Q5	0.60	
	Q7	0.91	
	Q8	0.83	
	Q9	0.80	
**Factor2: Subjective or Psychological** **fear**	Q3	0.55	20.4%
	Q10	0.61	
	Q11	0.65	
	Q12	0.75	
	Q13	0.69	

#### 3.4.2. Confirmatory Factor Analysis.

**Fig 2:**
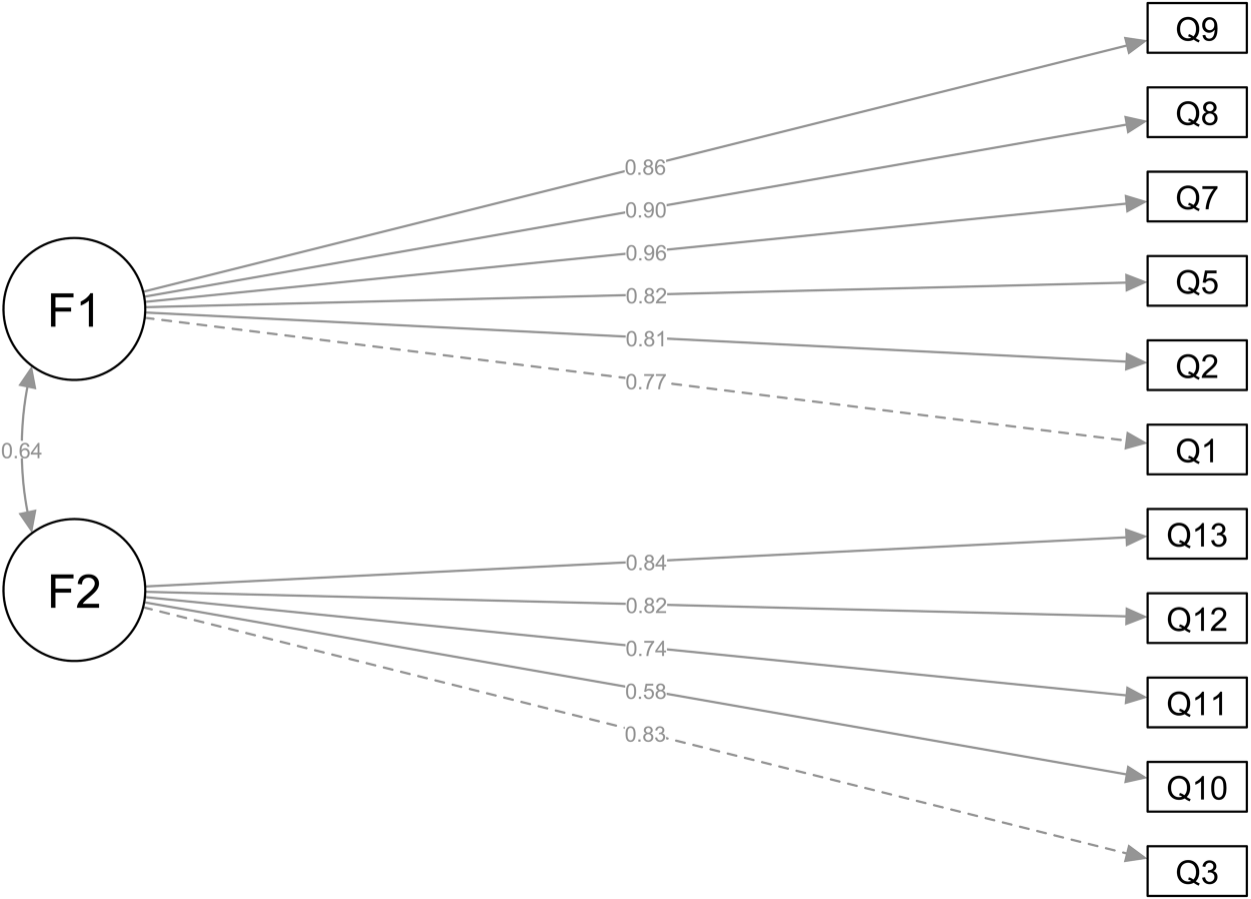
Confirmatory factor analysis model of the Persian version of the CheSeFS Scale (N = 300).

A CFA was conducted to evaluate the structural factors of the model. The findings validated all the goodness-of-fit indices for the final model. The results indicated that all model fit indices were within acceptable ranges, confirming the adequate fit of the two-dimensional model of the CheSeFS (Fig 2).

Specifically, the CFA model demonstrated a satisfactory fit to the data, with the following statistics: χ² (df = 43) = 65.8, p = 0.014; RMSEA = 0.04 (95% CI: 0.02–0.06); CFI = 1.00; TLI = 1.00; and SRMR = 0.04 (95% CI: 0.02–0.06). These metrics collectively confirmed the good fit of the extracted model.

The linear regression model demonstrated a good fit, with an R value of 0.743, explaining 55.2% of the variance in CheSeFS scores (R² = 0.552). The adjusted R² value of 0.543 indicated that the model remained stable after accounting for the number of predictors. Multicollinearity diagnostics showed acceptable variance inflation factor (VIF) values ranging from 1.05 to 1.85 and tolerance values between 0.54 and 0.96, indicating no evidence of problematic multicollinearity among predictors.

Among the predictors, higher pain scores were significantly associated with increased fear levels (β = 0.19, p < .001). Male participants reported higher fear scores than females (β = 0.96, p < .001), and participants with graduate-level education also showed higher scores (β = 0.90, p < .001). Urban residence was associated with higher fear scores (β = 1.52, p < .001), whereas being married was associated with lower scores (β = −0.21, p = .001). Additionally, chronic illness was positively associated with fear (β = 0.47, p < .001), while having a family history of cancer was linked to lower fear scores (β = −0.70, p < .001). Full regression details are presented in Table 4.

**Table 4:** Multiple linear regression analysis predicting CheSeFS scores (N = 600).

Predictor	β (Standardized Estimate)	95% CI		SE	t	P
		Lower 95% CI	Upper 95% CI			
**Intercept**	–	–	–	1.17	11.11	P < .001
**Age**	0.05	−0.01	0.11	0.13	1.53	P = 0.13
**Pain**	**0.19**	**0.13**	**0.24**	**0.09**	**6.68**	**P < .001**
**Gender:** Male – Female	**0.96**	**0.79**	**1.12**	**0.46**	**11.17**	**P < .001**
**Education level:** Graduate – Undergraduate	**0.90**	**0.63**	**1.17**	**0.74**	**6.53**	**P < .001**
**Marital status:** Married – Single	**−0.21**	**−0.33**	**−0.08**	**0.33**	**−3.29**	**P = 0.001**
**Region:** City – Village	**1.52**	**1.36**	**1.68**	**0.44**	**18.45**	**P < .001**
**Employment status:** Employed – Unemployed	−0.14	−0.29	−0.00	0.40	−1.92	P = 0.06
**Type of venous access:** Central-Peripheral	**−0.22**	**−0.36**	**−0.07**	**0.39**	**−2.97**	**P = 0.003**
**Smoking:** Yes-No	0.08	−0.05	0.22	0.37	1.20	P = 0.23
**Chronic Disease:** Yes – No	**0.47**	**0.34**	**0.61**	**0.37**	**6.91**	**P < .001**
**Cancer in Family:** Yes – No	**−0.70**	**−0.84**	**−0.55**	**0.39**	**−9.47**	**P < .001**

#### 3.4.3. Convergent and discriminant validity.

The CR values exceeded the recommended threshold of 0.70 for both factors (Factor 1 = 0.91; Factor 2 = 0.80), indicating adequate internal consistency. The AVE values were 0.73 for Factor 1 and 0.59 for Factor 2, both exceeding the recommended cutoff of 0.50, confirming satisfactory convergent validity.

The HTMT value between the two latent constructs was 0.60, which was below the recommended threshold of 0.85, indicating adequate discriminant validity and supporting the distinctiveness of the two-factor structure.

### 3.5. Reliability

The reliability of the CheSeFS was assessed through rigorous statistical analysis. Internal consistency was robust, as indicated by a Cronbach’s alpha coefficient of 0.89 and McDonald’s ω of 0.89 for the overall scale, demonstrating the coherence of the items within the measure. Additionally, the scale exhibited excellent test-retest reliability, as evidenced by an Intraclass Correlation Coefficient (ICC) of 0.89, suggesting high stability and consistency of the scores over time. Table 5 shows the results in detail.

**Table 5:** Reliability analysis of the CheSeFS scale.

Factor (Subscale)	Items	Cronbach’s α	McDonald’s ω	ICC (95%CI)
Factor1: Physiological fear	6	0.90	0.91	0.91 (0.82-0.96)
Factor2: Subjective or Psychological fear	5	0.79	0.80	0.86 (0.74-0.93
Total	11	0.89	0.89	0.89 (0.79-0.95)

### 3.6. Final adapted scale and scoring guide

The final adapted Persian CheSeFS contains 11 items distributed across two subscales. The Physiological fear subscale includes items 1, 2, 5, 7, 8, and 9, and the Subjective or Psychological fear subscale includes items 3, 10, 11, 12, and 13. Items 4, 6, and 14 were removed during factor analysis because they did not contribute to a stable and conceptually coherent two-factor structure.

Each item is scored on a 5-point Likert scale from 1 to 5, with higher scores indicating greater fear of chemotherapy-related side effects. The total score is calculated by summing all 11 items and ranges from 11 to 55. The Physiological fear subscale score ranges from 6 to 30, and the Subjective or Psychological fear subscale score ranges from 5 to 25. No items are reverse scored. The final adapted scale and scoring instructions are provided as S4 File.

## 4. Discussion

Fear of chemotherapy-induced side effects is a well-documented psychological concern among patients with cancer, which can significantly impair adherence to treatment regimens and ultimately compromise therapeutic outcomes ^45^. To the best of our knowledge, CheSeFS is among the few validated scales with a specific focus on chemotherapy-related fear. Given the profound implications of chemotherapy-related fear, the current study aimed to validate the Persian version of the CheSeFS, which can now be employed in both clinical and research settings within the Iranian population.

The results of the current study confirm the robust validity of the Persian version of the CheSeFS. The face validity analysis revealed that all items had an impact score greater than 1.5, leading to the conclusion that none of the items should be excluded from the scale. This supports the comprehensiveness of the scale in capturing the breadth of concerns regarding chemotherapy’s side effects. Furthermore, the content validity evaluation yielded a CVR of 1 for each item, meeting the acceptable threshold, and an I-CVI ranging from 0.80 to 1, indicating strong agreement among experts regarding the relevance and clarity of the items. These results are consistent with the findings of Ivana Vasić et al., who reported satisfactory face and content validity for the original version of the CheSeFS [15]. The alignment of the present findings with those from the original scale reinforces the robustness of the scale’s design and its relevance across different populations.

Beyond the quantitative evidence for face and content validity, the cognitive debriefing process provided important support for the cultural and practical suitability of the Persian CheSeFS. In cross-cultural adaptation studies, conceptual equivalence alone is not sufficient; the adapted scale must also be understandable, acceptable, and easy to complete for the target population. The patients’ feedback suggested that the Persian wording was appropriate for individuals undergoing chemotherapy and that the retained items were relevant to their treatment-related concerns. This strengthens the applicability of the scale in routine oncology and psycho-oncology settings.

EFA identified two distinct factors: (1) the physiological aspects of fear related to chemotherapy side effects and (2) the subjective aspects. These two factors accounted for 54.4% of the total variance, with factor loadings ranging from 0.53 to 0.85, which is indicative of satisfactory reliability. One item— “I am afraid of having to take antidepressant drugs”—was excluded from the final version due to a factor loading below the acceptable threshold. While this decision was methodologically justified, cultural factors may have contributed to its weak performance. In the Iranian context, the use of psychiatric medication may carry social stigma, potentially leading participants to underreport such fears. Moreover, discussions about antidepressant use may not be commonly integrated into oncology care, reducing the item’s salience. Although the item was removed, its underlying concept remains important, and future studies might consider alternative phrasing or contextual framing to better capture this dimension of fear. These findings align with previous studies by Vasić and Janković, who reported that the two-factor model explained 61.691% of the variance [15]. This consistency strengthens the argument for the CheSeFS’s generalizability and confirms the validity of its factor structure across cultural contexts.

CFA further supported the two-factor model, highlighting the multidimensional nature of chemotherapy-related fear, encompassing both physiological symptoms (e.g., nausea, alopecia, and fatigue) and psychological concerns (e.g., social isolation and anxiety related to treatment outcomes). These dimensions are consistent with existing literature that identifies chemotherapy side effects such as nausea, vomiting, pain, and alopecia as central to patients’ fears [34,35].

The final 11-item structure may also improve the practical usability of the Persian CheSeFS. Removing items that did not fit the final factor structure resulted in a more coherent and parsimonious scale while preserving the two main dimensions of chemotherapy-related fear. Providing a clear scoring guide is important for reproducibility and clinical interpretability, as it allows researchers and clinicians to calculate total and subscale scores consistently. This is particularly relevant for future studies that may compare fear levels across patient groups or evaluate changes in fear over time.

The present study’s findings also align with those reported by multiple authors, demonstrating that these side effects contribute significantly to patients’ psychological distress. Symptoms such as anorexia, hair loss, nausea, fatigue, and chronic diarrhea not only affect the physical health of patients with cancer but also foster anxiety, depression, and social withdrawal [36]. As noted by Nies et al., the fear of these side effects can lead to significant psychological distress, which often exacerbates the patient’s perception of their health condition and increases the likelihood of treatment non-compliance [34].

The findings of this study showed that several demographic and clinical variables were associated with CheSeFS scores among patients undergoing chemotherapy. Pain, education level, marital status, region of residence, employment status, type of venous access, chronic disease, family history of cancer, and gender were significantly associated with fear scores in the multivariable regression model. These findings should be interpreted as associations rather than causal relationships because of the cross-sectional design of the study. Therefore, the results identify patient characteristics that may help clinicians recognize groups with higher fear scores, but they do not establish that these variables cause changes in chemotherapy-related fear.

Pain was significantly associated with higher CheSeFS scores among patients undergoing chemotherapy. This association may reflect the physical and psychological burden experienced by patients who report pain during treatment. Pain may be perceived by some patients as a sign of treatment difficulty, disease progression, or uncertainty about treatment outcomes, which may be linked to higher levels of chemotherapy-related fear. However, because of the cross-sectional design of this study, this association should not be interpreted as evidence that pain causes greater fear. These findings suggest that patients reporting pain may require closer psychological assessment and supportive care during chemotherapy. This finding is consistent with previous evidence showing links between cancer-related fear, anxiety, and pain interference. This underscores the need for proactive pain management strategies in cancer care, including timely use of analgesics, psychological support, and patient education about pain control options.

Patients with graduate-level education reported significantly higher levels of fear compared to those with an undergraduate education. This finding aligns with the hypothesis that individuals with higher education levels may have greater awareness of the risks and potential complications of chemotherapy, leading to heightened fear. This finding aligns with the broader evidence suggesting that higher education levels, while contributing to better awareness and outcomes, may also heighten patients’ sensitivity to potential complications and risks, potentially leading to greater fear regarding treatment outcomes [37].

Marital status was also associated with CheSeFS scores, with married patients reporting lower fear scores compared with single patients. This result supports the role of emotional and social support in mitigating psychological distress. Married individuals may benefit from the emotional reassurance and practical assistance provided by their partners, which can reduce their fears about chemotherapy. Conversely, single patients may lack such support networks, leaving them more vulnerable to anxiety during treatment [38]. This finding aligns with prior research that highlights the protective role of social and emotional support in reducing fear of cancer. Lower levels of social support have been associated with higher fear, underscoring the importance of strong social networks in mitigating psychological distress during survivorship [39].

Urban residency was associated with higher fear scores compared with rural residency. While urban areas often provide better access to healthcare services, they are also associated with increased social and economic pressures, which may amplify fear about chemotherapy’s impact on patients’ personal and professional lives. Urban residents may also feel isolated in their treatment journey due to the fast-paced nature of city life [40]. While existing research has extensively documented rural-urban disparities in cancer outcomes [41], further studies are needed to explore the psychological and social impact of urban residency on patients undergoing chemotherapy.

Employment status showed a borderline association with CheSeFS scores, with employed patients reporting slightly higher fear scores compared with unemployed patients. The patients who are employed may experience additional stress related to balancing their professional responsibilities with the demands of cancer treatment. Concerns about job performance, income stability, and workplace expectations can exacerbate their fears. On the other hand, unemployed patients may feel less pressure in this regard, which could partially explain their lower fear levels [42]. This finding aligns with the results of a study on fear of cancer, which highlighted that employed individuals tend to experience higher levels of psychological distress due to work-related concerns during cancer survivorship [43].

Patients receiving chemotherapy through peripheral venous access reported significantly higher levels of fear compared to those with central venous access. Peripheral venous access is often associated with greater physical discomfort and a heightened perception of invasive procedures, which may contribute to increased anxiety [44]. This study aligns with our findings, demonstrating that patients undergoing peripheral venous access procedures often report heightened fear due to the discomfort and invasive nature of the procedure. The study highlights the need for effective communication and tailored interventions to address patients’ fears and improve their overall treatment experience [45].

Chronic disease was significantly associated with higher CheSeFS scores. Patients with chronic conditions may worry about the interaction between chemotherapy and their pre-existing illnesses, as well as their ability to cope with the cumulative health burden [46]. This study aligns with our findings, which highlight that patients with cancer with chronic diseases experience heightened emotional distress, particularly increased fear and anxiety [47].

Surprisingly, a family history of cancer was associated with significantly lower levels of fear. While direct studies exploring this relationship are limited, the potential protective effect may be attributed to the familiarity and emotional support provided by family members who have experienced cancer treatment themselves. Existing evidence highlights the importance of family support in alleviating emotional distress during cancer treatment, suggesting that a strong family support network can provide reassurance and reduce uncertainty [48]. These findings underscore the need for further research to explore this relationship and the role of family involvement in enhancing patients’ psychological well-being.

Gender differences were also significant, with male patients reporting higher levels of fear compared to female patients. This finding may reflect societal and cultural pressures that discourage men from openly expressing their emotions, leading to greater internalized fear [49]. Men may also be more concerned about the impact of chemotherapy on their professional and social roles, which could further exacerbate their fears [50]. These results are consistent with previous studies showing that men often report greater psychological distress related to cancer treatment compared to women [51,52].

Age was not a significant predictor of fear in this study. This finding aligns with previous research indicating that fear related to chemotherapy’s side effects may not vary substantially across age groups, as concerns about physical symptoms such as alopecia and nausea are common to all patients [53]. Similarly, smoking status was not a significant predictor, suggesting that fear of chemotherapy’s side effects is not influenced by smoking behavior [54].

The reliability findings further support the use of the Persian CheSeFS as a stable and internally consistent measure. The internal consistency results indicate that the retained items measure related aspects of chemotherapy-related fear without excessive heterogeneity. The test–retest findings also suggest that the scale produces stable scores over a short interval when no major change in the construct is expected. In addition, reporting measurement error improves the interpretability of score changes. The MDC95 provides a useful reference for distinguishing true change from random measurement error, which may be valuable in future longitudinal or intervention studies. In the present study, a distribution-based MIC estimate was also calculated to provide supportive evidence for score interpretability. The estimated MIC for the total CheSeFS score was 1.01 points using the small effect size criterion. However, this value was lower than the MDC95, indicating that small changes in CheSeFS scores may not exceed measurement error at the individual level. Therefore, this estimate should not be interpreted as an anchor-based or patient-perceived MIC, and the clinical meaningfulness of score changes should be further examined in future longitudinal studies.

### 4.1. Limitations

This study has several limitations that should be considered when interpreting the findings. First, the sample was limited to patients undergoing chemotherapy in a specific region of Iran, which may affect the generalizability of the results to other populations and cultural contexts. Future studies should include more diverse samples from different regions and clinical settings to ensure broader applicability of the findings.

Second, the cross-sectional design limits the ability to infer causal relationships between demographic and clinical variables and fear of chemotherapy-related side effects. Longitudinal studies are needed to explore these associations over time and to better understand the progression of fear during different stages of treatment.

In addition, although measurement error and a distribution-based MIC estimate were assessed, responsiveness and anchor-based MIC were not evaluated because the study did not include longitudinal change data, an external anchor, or patient-rated global change criteria. Therefore, the distribution-based MIC should be interpreted only as supportive evidence for score interpretability rather than as a definitive clinically meaningful change threshold. Future longitudinal studies should examine responsiveness and determine the smallest change in CheSeFS scores that is meaningful from the patient or clinical perspective.

Finally, although the Persian version of the CheSeFS demonstrated satisfactory psychometric properties, further validation in other populations, languages, and cultural settings is needed. Future studies should also examine the scale’s associations with external measures of related and unrelated psychological constructs to provide additional evidence of its external validity and clinical interpretability.

### 4.2. Practical implications

The findings of this study provide psychometric support for the Persian version of the CheSeFS as a scale for assessing chemotherapy-related fear among Iranian patients with cancer. The scale may be useful for identifying and describing the level and dimensions of fear related to chemotherapy side effects in clinical and research settings. Its two-subscale structure and scoring guide can help clinicians and researchers interpret physiological and subjective or psychological aspects of chemotherapy-related fear more consistently.

However, the present study did not evaluate any intervention, treatment adherence, psychological outcomes, or clinical outcomes. Therefore, the findings should not be interpreted as evidence that use of the CheSeFS reduces fear, improves adherence, or enhances patient outcomes. Future longitudinal and intervention studies are needed to determine whether assessment with the CheSeFS can inform supportive care strategies and whether such strategies affect chemotherapy-related fear, treatment adherence, psychological distress, or clinical outcomes.

### 5. Conclusion

This study translated, culturally adapted, and evaluated the psychometric properties of the Persian version of the CheSeFS among Iranian patients with cancer undergoing chemotherapy. The final 11-item Persian CheSeFS demonstrated satisfactory face validity, content validity, structural validity, convergent and discriminant validity, internal consistency, test–retest reliability, and measurement error indices. The scale includes two subscales representing physiological fear and subjective or psychological fear, with higher scores indicating greater chemotherapy-related fear.

Several demographic and clinical variables were associated with CheSeFS scores; however, these findings should be interpreted as associations rather than causal relationships because of the cross-sectional design. The present findings support the Persian CheSeFS as a psychometrically acceptable scale for assessing chemotherapy-related fear in this population. Future studies should examine its responsiveness, Minimal Important Change, and performance in longitudinal and intervention-based research.

## Supporting information

S1 Data De-identified dataset underlying the findings of the exploratory factor analysis. (SAV)

S2 Data De-identified dataset underlying the findings of the confirmatory factor analysis. (SAV)

S3 Data De-identified dataset underlying the findings of the test–retest reliability assessment (ICC calculation). (SAV)

S4 File Final adapted Persian version of the Chemotherapy Side Effects Fear Scale and its scoring guide. (PDF)
