# Supplementary material for: Psychometric properties of the Persian version of the chemotherapy side effects fear scale among Iranian patients with cancer
Source: PLoS One. 2026 Aug 13;21(8):e0354374. doi: 10.1371/journal.pone.0354374 (PMC13472437; doi:10.1371/journal.pone.0354374)
Supplement: Supplementary file 1 [file pone.0354374.s004.pdf]

پرسشنامه مقیاس ترس از عوارض شیمی درمانی

موارد زیر دلایل احتمالی را توضیح می‌دهند که می‌تواند باعث ترس شما از دریافت درمان شیمی‌درمانی شود. لطفاً هر مورد را با دقت مرور کنید و به این فکر کنید که این جملات چقدر احساس ترس در شما ایجاد می کنند. لطفاً دور گزینه ای که نمایانگر میزان ترس شما از عوارض جانبی شیمی درمانی مرتبط با هر مورد می باشد، دایره بکشید.

۱. از کاهش (ازدست دادن) اشتها، حس چشایی و بویایی به مدت طولانی می ترسم.

۱. هرگز، به هیچ وجه ۲. بعضی اوقات ۳. اغلب اوقات ۴. بیشتر اوقات ۵. همیشه

۲. از ریزش موهایم و استفاده از کلاه گیس میترسم.

۱. هرگز، به هیچ وجه ۲. بعضی اوقات ۳. اغلب اوقات ۴. بیشتر اوقات ۵. همیشه

۳. میترسم نتوانم دوستانم را ملاقات کنم و با آنها وقت بگذرانم.

۱. هرگز، به هیچ وجه ۲. بعضی اوقات ۳. اغلب اوقات ۴. بیشتر اوقات ۵. همیشه

۴. از احساس درد شدید در حین شیمی درمانی می ترسم.

۱. هرگز، به هیچ وجه ۲. بعضی اوقات ۳. اغلب اوقات ۴. بیشتر اوقات ۵. همیشه

۵. از کاهش وزن سریع میترسم.

۱. هرگز، به هیچ وجه ۲. بعضی اوقات ۳. اغلب اوقات ۴. بیشتر اوقات ۵. همیشه

۶. از اینکه مجبور شوم داروهای ضد افسردگی مصرف کنم می ترسم.

۱. هرگز، به هیچ وجه ۲. بعضی اوقات ۳. اغلب اوقات ۴. بیشتر اوقات ۵. همیشه

۷. از ایجاد زخم در دهان و گلودرد می ترسم.

۱. هرگز، به هیچ وجه ۲. بعضی اوقات ۳. اغلب اوقات ۴. بیشتر اوقات ۵. همیشه

۸. از آنمی (کم خونی)، تهوع و احساس خستگی می ترسم.

۱. هرگز، به هیچ وجه ۲. بعضی اوقات ۳. اغلب اوقات ۴. بیشتر اوقات ۵. همیشه

۹. از اسهال مداوم می ترسم.

۱. هرگز، به هیچ وجه ۲. بعضی اوقات ۳. اغلب اوقات ۴. بیشتر اوقات ۵. همیشه

۱۰. از اینکه با سایر بیماران منتظر درمان باشم می ترسم.

۱. هرگز، به هیچ وجه ۲. بعضی اوقات ۳. اغلب اوقات ۴. بیشتر اوقات ۵. همیشه

۱۱. از اختلال در خوابم و مشکل به خواب رفتن می ترسم.

۱. هرگز، به هیچ وجه ۲. بعضی اوقات ۳. اغلب اوقات ۴. بیشتر اوقات ۵. همیشه

۱۲. از ابتلا به عفونت های مقاوم و ضعیف شدن سیستم ایمنی ام می ترسم.

۱. هرگز، به هیچ وجه ۲. بعضی اوقات ۳. اغلب اوقات ۴. بیشتر اوقات ۵. همیشه

۱۳. از عدم دسترسی به اطلاعات تخصصی زمانی که نیاز دارم میترسم.

۱. هرگز، به هیچ وجه ۲. بعضی اوقات ۳. اغلب اوقات ۴. بیشتر اوقات ۵. همیشه

۱۴. از عوارض جانبی طولانی مدت (ماندگار) بر روی قلبم، ریه هام، کلیه هام و غیره میترسم.

۱. هرگز، به هیچ وجه ۲. بعضی اوقات ۳. اغلب اوقات ۴. بیشتر اوقات ۵. همیشه

Scoring guide

The final adapted Persian CheSeFS contains 11 items distributed across two subscales. The Physiological fear subscale includes items 1, 2, 5, 7, 8, and 9, and the Subjective or Psychological fear subscale includes items 3, 10, 11, 12, and 13. Items 4, 6, and 14 were removed during factor analysis because they did not contribute to a stable and conceptually coherent two-factor structure. Each item is scored on a 5-point Likert scale from 1 to 5, with higher scores indicating greater fear of chemotherapy-related side effects. The total score is calculated by summing all 11 items and ranges from 11 to 55. The Physiological fear subscale score ranges from 6 to 30, and the Subjective or Psychological fear subscale score ranges from 5 to 25. No items are reverse scored.
