# Supplementary material for: Psychometric properties of the Persian version of the chemotherapy side effects fear scale among Iranian patients with cancer
Source: PLoS One. 2026 Aug 13;21(8):e0354374. doi: 10.1371/journal.pone.0354374 (PMC13472437; doi:10.1371/journal.pone.0354374)
Supplement: Supplementary file 2 [file pone.0354374.s006.docx]

**Dear Editor and Reviewer**

Thank you for your letter and for the reviewer's comments concerning our manuscript entitled “Psychometric properties of the Persian version of the Chemotherapy Side Effects Fear Scale among Iranian cancer patients ". Those comments are all valuable and very helpful for revising and improving our paper, as well as providing important guidance for our research. We have carefully studied the comments and made corrections that we hope will meet with approval. The revised portion is marked with Track Changes in the paper. The main corrections in the paper and the responses to Editor and Reviewer comments are as follow:

=====================================================================

**1. Editor:**

**1.1 Comment:** Please ensure that your manuscript meets PLOS ONE's style requirements, including those for file naming. The PLOS ONE style templates can be found at
https://journals.plos.org/plosone/s/file?id=wjVg/PLOSOne_formatting_sample_main_body.pdf and
https://journals.plos.org/plosone/s/file?id=ba62/PLOSOne_formatting_sample_title_authors_affiliations.pdf

Response:

Thank you for pointing this out. We carefully made all corrections needed.

**1.2 Comment: We note that the grant information you provided in the ‘Funding Information’ and ‘Financial Disclosure’ sections do not match.
When you resubmit, please ensure that you provide the correct grant numbers for the awards you received for your study in the ‘Funding Information’ section.**

**Response:**

Thank you for pointing this out. We carefully reviewed the information provided in the Funding Information and Financial Disclosure sections and corrected the inconsistency. Both sections have now been revised to accurately state that this study was funded by the Nursing and Midwifery Care Research Center, Tehran University of Medical Sciences, Tehran, Iran, under grant number 57727.

**1.3 Comment: Thank you for stating the following financial disclosure:
“The author(s) received no specific funding for this work.”**

**At this time, please address the following queries:
a) Please clarify the sources of funding (financial or material support) for your study. List the grants or organizations that supported your study, including funding received from your institution.
b) State what role the funders took in the study. If the funders had no role in your study, please state: “The funders had no role in study design, data collection and analysis, decision to publish, or preparation of the manuscript.”
c) If any authors received a salary from any of your funders, please state which authors and which funders.
d) If you did not receive any funding for this study, please state: “The authors received no specific funding for this work.”
Please include your amended statements within your cover letter; we will change the online submission form on your behalf.**

**Response:**

Thank you for this clarification. We confirm that this study was funded by the Nursing and Midwifery Care Research Center, Tehran University of Medical Sciences, Tehran, Iran, under grant number 57727. The funders had no role in study design, data collection and analysis, decision to publish, or preparation of the manuscript. No authors received a salary from the funder for this study. We have included the amended financial disclosure statement in the cover letter as requested.

**1.4 Comment: Your ethics statement should only appear in the Methods section of your manuscript. If your ethics statement is written in any section besides the Methods, please delete it from any other section.**

**Response:**

Thank you for this comment. We have revised the manuscript accordingly. The ethics statement now appears only in the Methods section under “Ethical considerations.” The duplicate ethics approval and consent statement that appeared in the Declaration section has been removed from the revised manuscript.

**1.5 Comment: Thank you for providing your underlying data as Supporting Information. We note that the data set contains text or data that is not in English. Please note that PLOS is an English-language publisher, so we require data sets to be provided in English as well. Please upload an English-language version of your data set.
 This will also allow us to determine if your data follows PLOS standards per our Data Availability policy here:** [**https://journals.plos.org/plosone/s/data-availability**](https://journals.plos.org/plosone/s/data-availability)**.**

**Response:**

We have uploaded an English-language version of the de-identified dataset as Supporting Information file S1 Dataset. All variable names, value labels, category labels, and explanatory text have been translated into English.

**1.6 Comment:  Please include captions for your Supporting Information files at the end of your manuscript, and update any in-text citations to match accordingly. Please see our Supporting Information guidelines for more information: http://journals.plos.org/plosone/s/supporting-information.**

**Response:**

Thank you for this comment. We have added a Supporting Information caption at the end of the manuscript and updated the in-text citation and Data Availability statement accordingly. The English-language de-identified dataset is cited in the manuscript as “S1 Dataset,” and the caption has been added at the end of the manuscript as: “S1 Dataset. De-identified minimal dataset underlying the findings of the study.”

**1.7 Comment: If the reviewer comments include a recommendation to cite specific previously published works, please review and evaluate these publications to determine whether they are relevant and should be cited. There is no requirement to cite these works unless the editor has indicated otherwise.**

**Response:**

Thank you for this clarification. We carefully reviewed and evaluated the relevant previously published literature in relation to the aims, methodology, and discussion of the present study. Based on this evaluation, the reference list has been updated where scientifically appropriate. We did not add references solely on the basis of recommendation; only publications that were considered relevant to the manuscript were included.

=====================================================================

**Reviewer 1:**

**1.1 Comment: The abstract does not reference the specific model used for the translation of the instrument or define the key psychometric performance indicators employed in the evaluation.**

**Response:** We sincerely thank the reviewer for this valuable comment. As suggested, we have revised the abstract to clearly specify the translation model and the key psychometric performance indicators employed in the evaluation. The translation process is now explicitly described as a forward–backward translation and cross-cultural adaptation based on the Beaton et al. guidelines. Additionally, we clarified that the psychometric evaluation was conducted according to COSMIN standards and included assessments of face validity, content validity (CVR and CVI), construct validity through exploratory and confirmatory factor analyses, convergent and discriminant validity, internal consistency (Cronbach’s alpha and McDonald’s ω), and test–retest reliability (ICC).

**1.2 Comment: The source cited for cancer prevalence data appears outdated and should be updated. The authors disproportionately emphasize assessment tools for stress, depression, and anxiety over fear. Consequently, the rationale and necessity for the cross-cultural adaptation of a fear-specific assessment tool for the Iranian context are insufficiently articulated.**

**Response:**

Thank you for your valuable comment. The information related to cancer prevalence has been updated using the most recent available data, and the revised sections have been highlighted in the manuscript for clarity. Also, we have revised the Introduction to clarify the conceptual distinction between fear and general psychological distress and to emphasize that fear of chemotherapy side effects is a treatment-specific construct associated with clinically relevant outcomes. Accordingly, we have strengthened the rationale for the cross-cultural adaptation and validation of a fear-specific instrument for the Iranian context.

**1.3 Comment:** **The methodology section omits a reference to the specific translation model adopted (e.g., the World Health Organization guidelines or another established framework) and does not detail its distinct phases. Only five generic steps are listed without citing a credible, verifiable source to substantiate this approach. Furthermore, the type of Content Validity Ratio (CVR) calculation applied (i.e., whether relaxed or strict criteria were used) and the method for calculating the Scale Content Validity Index (SCVI) are not clearly specified. The procedures for measuring the agreement coefficient (Kappa) and conducting item analysis before factor analysis are also not described.**

**Response:**

Thank you for this important comment. The Methodology section has been revised to explicitly state that the translation and cross-cultural adaptation process was conducted in accordance with the guidelines proposed by Beaton et al. The distinct phases of the model (forward translation, synthesis, back translation, expert committee review, pre-testing, and documentation) are now clearly described, and the appropriate reference has been added to substantiate the adopted framework. The Methodology section has been revised to explicitly describe the CVR calculation based on Lawshe’s strict criterion, the computation of the Item-level Content Validity Index (I-CVI), and the procedures for item analysis before exploratory factor analysis, including corrected item–total correlations and distributional indices. These additions clarify the statistical procedures applied in the validation process.

**1.4 Comment:** **The findings provide an inadequate account of the modification or revision of terms, items, and cultural adaptations during the assessment of face validity, content validity, and item analysis. Additionally, the procedure for administering the preliminary test to establish sample adequacy for confirmatory factor analysis is not reported. The calculations for the Minimal Important Change (MIC), Minimal Detectable Change (MDC), and the tests for ceiling/floor effects and response ease are not clearly delineated. The presentation of the final, adapted instrument and a clear scoring guide is also lacking.**

**Response:**

Thank you for this valuable comment. We revised the manuscript to provide a clearer description of the cross-cultural adaptation process, face validity, content validity, preliminary item analysis, CFA sample adequacy, measurement error, and scoring procedure.

We added details on the expert committee review, cognitive interviews, cultural appropriateness, response ease, and minor wording revisions made to improve clarity and naturalness of the Persian version. We clarified that no item was added or removed during the translation, face validity, or content validity stages.

We also added a new subsection on preliminary item analysis and measurement error. Corrected item–total correlations ranged from 0.383 to 0.783, all exceeding the 0.30 criterion. Missing or incomplete responses were minimal. No substantial floor or ceiling effects were observed, as all values were below the predefined 15% threshold.

Measurement error was also reported. Based on the test–retest data, SEM was 1.64 and MDC95 was 4.56.

Measurement error was also reported. Based on the test–retest data, the SEM was 1.64 and the MDC95 was 4.56. Because the study did not include a longitudinal external anchor or patient-rated global change criterion, an anchor-based MIC was not estimated. However, to provide supportive evidence for score interpretability, a distribution-based MIC estimate was calculated using the small effect size criterion. The distribution-based MIC estimate for the total CheSeFS score was 1.01 points.

We further clarified that CFA was conducted on an independent sample of 300 participants. Considering the final 11-item structure, this provided more than 25 participants per retained item, supporting sample adequacy for CFA.

Finally, we added a section presenting the final adapted 11-item Persian CheSeFS and its scoring guide. The final instrument and scoring instructions have also been provided as Supporting Information file S2 File.

**1.5 Comment: While the discussion is consistent with the reported findings, addressing the deficiencies identified in the methodology and results would necessitate a corresponding enrichment of the discussion section.**

**Response:**

Thank you for this helpful comment. We enriched the Discussion section to reflect the methodological and results-related revisions. Specifically, we added discussion of cognitive debriefing, cultural appropriateness, response ease, item homogeneity, floor and ceiling effects, measurement error indices including SEM and MDC95, the supportive distribution-based MIC estimate, and the implications of the final 11-item adapted scale and scoring guide. We also clarified the interpretation of score change by noting that the distribution-based MIC estimate should not be interpreted as an anchor-based or patient-perceived MIC, because longitudinal change data, an external anchor, and patient-rated global change criteria were not collected. The Limitations section was also revised to state that responsiveness and anchor-based MIC should be examined in future longitudinal studies.

=====================================================================

**Reviewer 2:**

**2.1 Comment:** **The manuscript repeatedly uses the term “cancer patients”, which reflects a labeling-based approach. Accordingly, the authors are encouraged to replace this terminology with “patients with cancer” throughout the manuscript. This revision would improve the ethical tone of the paper and align it with internationally accepted standards for respectful and person-centered scientific communication.**

**Response:**

Thank you for your thorough feedback. All requested revisions have been implemented.

**2.2 Comment:** **The study is cross-sectional, yet predictors are discussed in quasi-causal language.**

**Response:**

Thank you for this important comment. We agree that, because of the cross-sectional design of the study, the regression findings should be interpreted as associations rather than causal relationships. We revised the Discussion and Conclusion sections accordingly. Specifically, we replaced quasi-causal terms such as “predictors,” “shaping,” “leading to,” “amplifying,” and “reducing” with more cautious language such as “variables associated with,” “may be associated with,” and “linked to.” We also added a clear statement that the identified demographic and clinical variables should not be interpreted as causes of chemotherapy-related fear. In addition, we noted that future longitudinal studies are needed to clarify temporal and causal relationships.

**2.3 Comment: The authors claim that the instrument was evaluated based on COSMIN criteria; however, key COSMIN domains—particularly measurement error—were not assessed or reported. If COSMIN is cited as the guiding framework, all relevant domains should be systematically evaluated and documented, or the claim should be revised accordingly.**

**Response:**

Thank you for this important comment. We revised the manuscript to avoid implying that all COSMIN domains were fully evaluated. The text now states that the selection and reporting of measurement properties were guided by relevant COSMIN recommendations.

We also added measurement error analysis to the Methods and Results sections, including the standard error of measurement (SEM) and the minimal detectable change at the 95% confidence level (MDC95). The SEM and MDC95 for the total CheSeFS score were 1.64 and 4.56, respectively. In addition, to provide supportive evidence for score interpretability, we calculated a distribution-based MIC estimate using the small effect size criterion, resulting in an estimated MIC of 1.01 points for the total score.

We further clarified that an anchor-based MIC, responsiveness, and criterion validity were not evaluated because the study did not include longitudinal change data, an external anchor, patient-rated global change criteria, or an appropriate gold standard. This limitation has also been addressed in the Discussion and Limitations sections.

**2.4 Comment: There is inconsistency in the terminology used to describe the scale (e.g., tool, instrument, questionnaire, inventory). The authors should standardize terminology throughout the manuscript to avoid confusion.**

**Response:**

Thank you for this comment. We revised the manuscript to standardize the terminology used to describe the CheSeFS. The term “scale” is now used consistently throughout the manuscript when referring to the Chemotherapy Side Effects Fear Scale. Inconsistent terms such as “tool,” “instrument,” “questionnaire,” and “inventory” were replaced where appropriate to avoid confusion.

**2.5 Comment: The abstract does not clearly report the time of study and the statistical software packages used for each analysis (e.g., SPSS, AMOS). Using the abbreviations CVR and CVI in an abstract is not common.**

**Response**:

Thank you for this helpful comment. We revised the Abstract to include the study period and the statistical software used for the analyses. We clarified that all statistical analyses were performed using Jamovi version 2.7.1. We also removed the abbreviations CVR and CVI from the Abstract and replaced them with a general statement indicating that patient and expert evaluations supported face and content validity.

**2.6 Comment: The keywords and abstract require revision. Additionally, keywords should be aligned with MeSH or commonly accepted psychometric terminology. In particular, the phrase “Validity and Reliability” should be standardized and corrected.**

**Response:**

Thank you for this helpful comment. We revised the Abstract to improve clarity and to include the study period and the statistical software used for the analyses. We also removed the abbreviations CVR and CVI from the Abstract and replaced them with a general statement that patient and expert evaluations supported face and content validity. In addition, the keywords were revised and standardized using MeSH-aligned and commonly accepted psychometric terminology. Specifically, “Validity and Reliability” was replaced with “Reproducibility of Results,” and the keyword list was revised to better reflect the study focus.

**2.7 Comment: Several sections include didactic or textbook-style explanations of basic psychometric concepts that are unnecessary for a scientific audience. For example: “Factor loading refers to the relationship between each factor …”. Such explanatory statements should be removed to improve conciseness and scholarly tone.**

**Response:**

Thank you for this helpful comment. We revised the manuscript to improve conciseness and scholarly tone by removing textbook-style explanations of basic psychometric concepts. The Methods section now focuses on the procedures and criteria used in the study rather than general methodological definitions.

**2.8 Comment: Maximum Likelihood extraction is used together with Varimax (orthogonal) rotation, although the two factors are conceptually correlated. CFA later implicitly assumes correlated latent factors. This is statistically inconsistent. An oblique rotation (e.g., Promax or Oblimin) would have been more appropriate.**

**Response:**

Thank you for this valuable observation. We agree that the use of orthogonal rotation (Varimax) may not be appropriate when latent factors are conceptually expected to be correlated. Accordingly, the exploratory factor analysis was re-conducted using maximum likelihood extraction with an oblique rotation (Promax), which allows correlations among factors. This modification ensured methodological consistency with the subsequent confirmatory factor analysis, where correlated latent constructs were specified. The revised analysis yielded a stable and interpretable factor structure, and the manuscript has been updated accordingly.

**2.9 Comment: The decision on factor retention relied only on Eigenvalues > 1and Scree plot, and Parallel analysis was not performed, which is now considered a minimum standard in psychometric research.**

**Response:**

Thank you for this important comment. We agree that relying solely on the Kaiser criterion (Eigenvalues > 1) and the scree plot may not provide the most robust basis for factor retention. In response to this suggestion, we re-conducted the exploratory factor analysis and incorporated parallel analysis to determine the optimal number of factors. Parallel analysis supported the retention of a two-factor solution, which was also consistent with theoretical expectations and the scree plot inspection. The manuscript has been revised to reflect this methodological improvement.

**2.10 Comment: RMSEA = 0.08 is interpreted as acceptable, although it is at the upper limit and often considered marginal, especially with large samples. Confidence intervals for RMSEA are not reported.**

**Response:**

Thank you for this helpful comment. We agree that RMSEA values close to 0.08 should be interpreted cautiously, particularly in larger samples. In response, we have revised the manuscript to report the 95% confidence interval for RMSEA and to provide a more balanced interpretation of model fit. The updated CFA results show a scaled RMSEA of 0.056 (95% CI: 0.044–0.067), indicating good model fit in the revised model. The corresponding revisions have been incorporated into the Results section.

**3.11 Comment: No modification indices or correlated error terms are discussed, despite borderline fit indices. Provide a more cautious and transparent interpretation of model fit and report full CFA parameters.**

**Response:**

Thank you for this valuable comment. In response, we re-ran the CFA following standard analytical procedures and model specification. The revised analysis yielded improved model fit indices, including satisfactory RMSEA, SRMR, CFI, and TLI values, supporting the adequacy of the proposed factor structure.

Given the acceptable model fit obtained in the revised analysis, additional post-hoc modifications such as introducing correlated error terms or relying on modification indices were not deemed necessary. The manuscript has been updated to provide a more cautious interpretation of model fit and to report the complete set of CFA parameters.

**3.12 Comment: The model explains over 50% of variance (R² = 0.516), which is unusually high for psychosocial constructs and raises concerns about Multicollinearity, Overfitting, and conceptual overlap between predictors and outcome.**

**Response:**

Thank you for this thoughtful comment. We acknowledge that relatively high explained variance in psychosocial research requires careful evaluation. In the present study, the regression model explained 55.2% of the variance in CheSeFS scores (Adjusted R² = 0.543). To assess potential multicollinearity, collinearity diagnostics were examined, showing variance inflation factor (VIF) values ranging from 1.05 to 1.85 and tolerance values well above recommended thresholds, indicating no problematic multicollinearity.

Additionally, the adjusted R² value was close to the observed R², suggesting that the model was not overfitted despite the number of predictors. The relatively large sample size (N = 600) compared with the number of predictors further supports model stability. Moreover, the predictors consisted of conceptually distinct demographic and clinical characteristics, reducing concerns about conceptual overlap with the outcome measure.

**3.13 Comment: Cronbach’s alpha and McDonald’s ω are reported as identical (0.90), which is uncommon and suggests possible rounding or reporting errors. Please report CR (composite reliability).**

**Response:**

Thank you for this valuable observation. We agree that Cronbach’s alpha and McDonald’s ω are typically not identical reliability estimates. In our initial reporting, rounding contributed to their apparent similarity. To improve transparency and provide a model-based reliability estimate, we have now reported composite reliability (CR) derived from the CFA model in addition to Cronbach’s alpha and omega coefficients. These values provide a more accurate representation of the internal consistency of the constructs and have been added to the revised manuscript.

**3.14 Comment: The study does not report Average Variance Extracted (AVE) or Heterotrait–Monotrait Ratio (HTMT), which are standard indicators for assessing convergent and discriminant validity in construct validation studies. Their absence weakens the evidence for construct validity.**

**Response:**

Thank you for this important comment. We agree that reporting AVE and HTMT strengthens the assessment of convergent and discriminant validity in construct validation studies. In response, we have now calculated and reported both AVE and HTMT values in the revised manuscript.

**3.15 Comment: ICC values are extremely high (up to 0.98) based on only 30 participants, which may inflate stability estimates. Please justify the test–retest sample size and discuss potential inflation of ICC.**

**Response:**

Thank you for this important comment. The test–retest reliability was assessed using a subsample of 30 participants. Although a larger retest sample could increase the precision of ICC estimates, a sample size of approximately 30 participants is generally considered acceptable for preliminary stability assessment in psychometric validation studies (Streiner & Norman, 2008).

In the present study, conducting a larger follow-up assessment was challenging due to the clinical condition of patients undergoing chemotherapy and the practical limitations in ensuring their return after a two-week interval. Nevertheless, ICC values were reported along with 95% confidence intervals to enhance transparency and provide precision estimates.

While we acknowledge that smaller retest samples may potentially yield less stable estimates, the high ICC values observed likely reflect the temporal stability of the construct rather than inflation. This limitation has now been explicitly discussed in the revised manuscript.

**3.16 Comment: The manuscript reports the Standardized Root Mean Square Residual (SRMR) as a model fit index in CFA. However, AMOS does not directly provide SRMR by default. The authors must clearly explain how SRMR was calculated, including whether an external plugin or additional computation was used.**

**Response:**

Thank you for this careful observation. We would like to clarify that the confirmatory factor analysis (CFA) in the revised manuscript was conducted using Jamovi software (version 2.7.1) with the SEM module based on the lavaan package, rather than AMOS. This software directly provides SRMR as part of the standard CFA output; therefore, no external plugin or additional computation was required. The manuscript has been revised to clarify the software used for CFA and the source of the reported fit indices.

**3.17 Comment: Although multicollinearity is mentioned, the manuscript does not report Variance Inflation Factor (VIF) and tolerance values in the regression table.**

**Response:**

Thank you for this insightful comment. Multicollinearity diagnostics were conducted as part of the regression assumption checks. The variance inflation factor (VIF) values ranged from 1.05 to 1.85, and tolerance values ranged from 0.54 to 0.96, indicating no evidence of problematic multicollinearity among the predictors. To improve transparency, these values have now been explicitly reported in the Results section of the revised manuscript.

**3.18 Comment: It is unclear whether age was originally collected as a categorical variable or whether it was categorized post hoc during analysis. If age was not grouped at the data collection stage, this should be clarified and corrected in the Methods section.**

**Response:**

Thank you for this important clarification request. Both age and pain were originally collected as continuous variables during data collection. In the revised manuscript, these variables are consistently reported in their original continuous form in Table 1 (Socio-demographic and Clinical Status Characteristics of the Participants).

**3.19 Comment: The abbreviations EFA and CFA are used for the first time in the Sample Size section without first providing their full forms.**

**Response:**

Thank you for this careful observation. The full forms of the abbreviations exploratory factor analysis (EFA) and confirmatory factor analysis (CFA) have already been introduced earlier in Section 2.1 (Study Design and Settings). However, to improve clarity and avoid any possible confusion, we have reviewed the manuscript and ensured that the abbreviations are clearly defined at their first occurrence.

**3.20 Comment: The manuscript repeatedly claims that the scale can guide tailored interventions/ It can improve treatment adherence and outcome; However, no intervention, longitudinal data, or adherence measures were assessed. These claims go beyond validation and are speculative. Limit conclusions strictly to psychometric properties.**

**Response:**

Thank you for this important comment. We agree that the previous wording went beyond the scope of a psychometric validation study. We revised the Practical Implications and Conclusion sections to remove speculative claims about tailored interventions, treatment adherence, and clinical outcomes. The revised manuscript now limits the interpretation to the psychometric properties of the Persian CheSeFS and states that future longitudinal and intervention studies are needed to examine whether use of the scale can inform supportive care or affect adherence, psychological distress, or clinical outcomes.

**3.21 Comment: Numerous grammatical and typographical errors reduce readability.**

**Response:**

Thank you for this comment. We carefully proofread and revised the manuscript to improve readability. Grammatical errors, typographical errors, punctuation, spacing, capitalization, section numbering, and inconsistent terminology were corrected throughout the manuscript. We also reviewed the revised text to ensure clearer sentence structure and smoother readability.

**3.22 Comment: References contain inconsistencies and irrelevant citations.**

**Response:**
Thank you for this helpful comment. We carefully reviewed the reference list and all in-text citations throughout the manuscript. Inconsistencies in formatting and citation details were corrected, and irrelevant or weakly related citations were removed or replaced with more appropriate and up-to-date references. The revised reference list is now consistent with the journal’s required style, and all cited sources are directly relevant to the corresponding statements in the manuscript.

**3.23 Comment: Tables are poorly aligned and sometimes duplicated conceptually in the text.**

**Response:**

Thank you for this helpful comment. The tables have been carefully reformatted to improve alignment, consistency, and overall presentation. In addition, the manuscript text has been revised to reduce conceptual duplication with the tables. The narrative sections now emphasize key findings and interpretations rather than repeating detailed numerical information that is already presented in the tables. These revisions were made to enhance readability and clarity throughout the manuscript.

**3.24 Comment: Some statistical thresholds are cited without references.**

**Response:**

Thank you for this valuable comment. We agree that all statistical thresholds should be supported by appropriate references. In the revised manuscript, we have added relevant citations for all reported cut-off criteria, including thresholds for model fit indices (e.g., RMSEA, CFI, TLI, SRMR), reliability coefficients (Cronbach’s α, Composite Reliability), convergent validity (AVE), discriminant validity (HTMT), and factor loading criteria. These references have been incorporated into the Methods section to ensure methodological transparency and alignment with established psychometric standards.

**2.25 Comment: Please report the mean and SD for all items and each factor.**

Response:

Thank you for this helpful comment. In response, we have now calculated and reported the mean and standard deviation for all individual items as well as for each extracted factor. In addition, to provide a more comprehensive evaluation of the data distribution, skewness and kurtosis indices have also been computed and included in the Results section. These statistics are presented in the newly added descriptive table to enhance transparency and completeness of reporting.
