# Supplementary material for: Psychometric properties of the Persian version of the chemotherapy side effects fear scale among Iranian patients with cancer
Source: PLoS One. 2026 Aug 13;21(8):e0354374. doi: 10.1371/journal.pone.0354374 (PMC13472437; doi:10.1371/journal.pone.0354374)
Supplement: Peer review history

# Peer review: Decision Letter 0

**DOI:** 10.1371/journal.pone.0354374.r001

**Authors:** Fatemeh Zarei

27 Jan 2026

Dear Dr. Negarrandeh

Thank you for submitting your manuscript to PLOS ONE. After careful consideration, we feel that it has merit but does not fully meet PLOS ONE’s publication criteria as it currently stands. Therefore, we invite you to submit a revised version of the manuscript that addresses the points raised during the review process.

==============================

Please submit your revised manuscript by Mar 13 2026 11:59PM. If you will need more time than this to complete your revisions, please reply to this message or contact the journal office at plosone@plos.org. When you're ready to submit your revision, log on to https://www.editorialmanager.com/pone/ and select the 'Submissions Needing Revision' folder to locate your manuscript file.

- A letter that responds to each point raised by the academic editor and reviewer(s). You should upload this letter as a separate file labeled 'Response to Reviewers'.
- A marked-up copy of your manuscript that highlights changes made to the original version. You should upload this as a separate file labeled 'Revised Manuscript with Track Changes'.
- An unmarked version of your revised paper without tracked changes. You should upload this as a separate file labeled 'Manuscript'.

If you would like to make changes to your financial disclosure, please include your updated statement in your cover letter. Guidelines for resubmitting your figure files are available below the reviewer comments at the end of this letter.

If applicable, we recommend that you deposit your laboratory protocols in protocols.io to enhance the reproducibility of your results. Protocols.io assigns your protocol its own identifier (DOI) so that it can be cited independently in the future. For instructions see: https://journals.plos.org/plosone/s/submission-guidelines#loc-laboratory-protocols. Additionally, PLOS ONE offers an option for publishing peer-reviewed Lab Protocol articles, which describe protocols hosted on protocols.io. Read more information on sharing protocols at https://plos.org/protocols?utm_medium=editorial-email&utm_source=authorletters&utm_campaign=protocols.

We look forward to receiving your revised manuscript.

Kind regards,

Fatemeh Zarei, PhD

Academic Editor

PLOS One

Journal Requirements:

When submitting your revision, we need you to address these additional requirements.

1. Please ensure that your manuscript meets PLOS ONE's style requirements, including those for file naming. The PLOS ONE style templates can be found at

https://journals.plos.org/plosone/s/file?id=wjVg/PLOSOne_formatting_sample_main_body.pdf and

https://journals.plos.org/plosone/s/file?id=ba62/PLOSOne_formatting_sample_title_authors_affiliations.pdf

2. We note that the grant information you provided in the ‘Funding Information’ and ‘Financial Disclosure’ sections do not match.

When you resubmit, please ensure that you provide the correct grant numbers for the awards you received for your study in the ‘Funding Information’ section.

3. Thank you for stating the following financial disclosure:

“The author(s) received no specific funding for this work.”

At this time, please address the following queries:

a) Please clarify the sources of funding (financial or material support) for your study. List the grants or organizations that supported your study, including funding received from your institution.

b) State what role the funders took in the study. If the funders had no role in your study, please state: “The funders had no role in study design, data collection and analysis, decision to publish, or preparation of the manuscript.”

c) If any authors received a salary from any of your funders, please state which authors and which funders.

d) If you did not receive any funding for this study, please state: “The authors received no specific funding for this work.”

Please include your amended statements within your cover letter; we will change the online submission form on your behalf.

4. Your ethics statement should only appear in the Methods section of your manuscript. If your ethics statement is written in any section besides the Methods, please delete it from any other section.

5. Thank you for providing your underlying data as Supporting Information.

We note that the data set contains text or data that is not in English. Please note that PLOS is an English-language publisher, so we require data sets to be provided in English as well. Please upload an English-language version of your data set.

This will also allow us to determine if your data follows PLOS standards per our Data Availability policy here: https://journals.plos.org/plosone/s/data-availability

6.  Please include captions for your Supporting Information files at the end of your manuscript, and update any in-text citations to match accordingly. Please see our Supporting Information guidelines for more information: http://journals.plos.org/plosone/s/supporting-information.

7. If the reviewer comments include a recommendation to cite specific previously published works, please review and evaluate these publications to determine whether they are relevant and should be cited. There is no requirement to cite these works unless the editor has indicated otherwise.

[Note: HTML markup is below. Please do not edit.]

Reviewers' comments:

Reviewer's Responses to Questions

**Comments to the Author**

1. Is the manuscript technically sound, and do the data support the conclusions?

Reviewer #1: Yes

Reviewer #2: Partly

**********

2. Has the statistical analysis been performed appropriately and rigorously?

Reviewer #1: No

Reviewer #2: No

**********

3. Have the authors made all data underlying the findings in their manuscript fully available?

The PLOS Data policy

Reviewer #1: Yes

Reviewer #2: Yes

**********

4. Is the manuscript presented in an intelligible fashion and written in standard English?

Reviewer #1: Yes

Reviewer #2: No

**********

Reviewer #1: Dear Editor-in-Chief of PLoS ONE,

Greetings.

Regarding the manuscript titled, "Psychometric properties of the Persian version of the Chemotherapy Side Effects Fear Scale among Iranian cancer patients,"

I offer the following feedback:

Abstract:

Abstract

The abstract does not reference the specific model used for the translation of the instrument or define the key psychometric performance indicators employed in the evaluation.

Introduction

The source cited for cancer prevalence data appears outdated and should be updated. The authors disproportionately emphasize assessment tools for stress, depression, and anxiety over fear. Consequently, the rationale and necessity for the cross-cultural adaptation of a fear-specific assessment tool for the Iranian context are insufficiently articulated.

Methods

The methodology section omits a reference to the specific translation model adopted (e.g., the World Health Organization guidelines or another established framework) and does not detail its distinct phases. Only five generic steps are listed without citing a credible, verifiable source to substantiate this approach. Furthermore, the type of Content Validity Ratio (CVR) calculation applied (i.e., whether relaxed or strict criteria were used) and the method for calculating the Scale Content Validity Index (SCVI) are not clearly specified. The procedures for measuring the agreement coefficient (Kappa) and conducting item analysis prior to factor analysis are also not described.

Results

The findings provide an inadequate account of the modification or revision of terms, items, and cultural adaptations during the assessment of face validity, content validity, and item analysis. Additionally, the procedure for administering the preliminary test to establish sample adequacy for confirmatory factor analysis is not reported. The calculations for the Minimal Important Change (MIC), Minimal Detectable Change (MDC), and the tests for ceiling/floor effects and response ease are not clearly delineated. The presentation of the final, adapted instrument and a clear scoring guide is also lacking.

Discussion

While the discussion is consistent with the reported findings, addressing the deficiencies identified in the methodology and results would necessitate a corresponding enrichment of the discussion section.

Reviewer #2: Dear Editor,

Thanks for inviting me to review this manuscript. This manuscript aims to validate the Persian version of the Chemotherapy Side Effects Fear Scale (CheSeFS) in Iranian cancer patients. The topic is relevant, and the sample size is relatively large. However, there are several major methodological, analytical, and reporting inconsistencies that substantially weaken the credibility of the findings.

1. The manuscript repeatedly uses the term “cancer patients”, which reflects a labeling-based approach. Accordingly, the authors are encouraged to replace this terminology with “patients with cancer” throughout the manuscript. This revision would improve the ethical tone of the paper and align it with internationally accepted standards for respectful and person-centered scientific communication.

2. The study is cross-sectional, yet predictors are discussed in quasi-causal language.

3. The authors claim that the instrument was evaluated based on COSMIN criteria; however, key COSMIN domains—particularly measurement error—were not assessed or reported. If COSMIN is cited as the guiding framework, all relevant domains should be systematically evaluated and documented, or the claim should be revised accordingly.

4. There is inconsistency in the terminology used to describe the scale (e.g., tool, instrument, questionnaire, inventory). The authors should standardize terminology throughout the manuscript to avoid confusion.

5. The abstract does not clearly report the time of study and the statistical software packages used for each analysis (e.g., SPSS, AMOS). Using the abbreviations CVR and CVI in an abstract is not common.

6. The keywords and abstract require revision. Additionally, keywords should be aligned with MeSH or commonly accepted psychometric terminology. In particular, the phrase “Validity and Reliability” should be standardized and corrected.

7. Several sections include didactic or textbook-style explanations of basic psychometric concepts that are unnecessary for a scientific audience. For example: “Factor loading refers to the relationship between each factor …”. Such explanatory statements should be removed to improve conciseness and scholarly tone.

8. Maximum Likelihood extraction is used together with Varimax (orthogonal) rotation, although the two factors are conceptually correlated. CFA later implicitly assumes correlated latent factors. This is statistically inconsistent. An oblique rotation (e.g., Promax or Oblimin) would have been more appropriate.

9. The decision on factor retention relied only on Eigenvalues > 1and Scree plot, and Parallel analysis was not performed, which is now considered a minimum standard in psychometric research.

10. RMSEA = 0.08 is interpreted as acceptable, although it is at the upper limit and often considered marginal, especially with large samples. Confidence intervals for RMSEA are not reported.

11. No modification indices or correlated error terms are discussed, despite borderline fit indices. Provide a more cautious and transparent interpretation of model fit and report full CFA parameters.

12. The model explains over 50% of variance (R² = 0.516), which is unusually high for psychosocial constructs and raises concerns about Multicollinearity,Overfitting, and conceptual overlap between predictors and outcome.

13. Cronbach’s alpha and McDonald’s ω are reported as identical (0.90), which is uncommon and suggests possible rounding or reporting errors. Please report CR (composite reliability).

14. The study does not report Average Variance Extracted (AVE) or Heterotrait–Monotrait Ratio (HTMT), which are standard indicators for assessing convergent and discriminant validity in construct validation studies. Their absence weakens the evidence for construct validity.

15. ICC values are extremely high (up to 0.98) based on only 30 participants, which may inflate stability estimates. Please justify the test–retest sample size and discuss potential inflation of ICC.

16. The manuscript reports the Standardized Root Mean Square Residual (SRMR) as a model fit index in CFA. However, AMOS does not directly provide SRMR by default. The authors must clearly explain how SRMR was calculated, including whether an external plugin or additional computation was used.

17. Although multicollinearity is mentioned, the manuscript does not report Variance Inflation Factor (VIF) and tolerance values in the regression table.

18. It is unclear whether age was originally collected as a categorical variable or whether it was categorized post hoc during analysis. If age was not grouped at the data collection stage, this should be clarified and corrected in the Methods section.

19. The abbreviations EFA and CFA are used for the first time in the Sample Size section without first providing their full forms.

20. The manuscript repeatedly claims that the scale can guide tailored interventions/ It can improve treatment adherence and outcome; However, no intervention, longitudinal data, or adherence measures were assessed. These claims go beyond validation and are speculative. Limit conclusions strictly to psychometric properties.

21. Numerous grammatical and typographical errors reduce readability.

22. References contain inconsistencies and irrelevant citations.

23. Tables are poorly aligned and sometimes duplicated conceptually in the text.

24. Some statistical thresholds are cited without references.

25. Please report the mean and SD for all items and each factor.

**********

what does this mean?). If published, this will include your full peer review and any attached files.

If you choose “no”, your identity will remain anonymous but your review may still be made public.

**Do you want your identity to be public for this peer review?**  For information about this choice, including consent withdrawal, please see our Privacy Policy

Reviewer #1: **Yes:** Fazlollah Ahmadi

Reviewer #2: **Yes:** Pardis Rahmatpour

**********

[NOTE: If reviewer comments were submitted as an attachment file, they will be attached to this email and accessible via the submission site. Please log into your account, locate the manuscript record, and check for the action link "View Attachments". If this link does not appear, there are no attachment files.]

To ensure your figures meet our technical requirements, please review our figure guidelines: https://journals.plos.org/plosone/s/figures

You may also use PLOS’s free figure tool, NAAS, to help you prepare publication quality figures: https://journals.plos.org/plosone/s/figures#loc-tools-for-figure-preparation.

NAAS will assess whether your figures meet our technical requirements by comparing each figure against our figure specifications.

# Author comment: Author response to Decision Letter 1

**DOI:** 10.1371/journal.pone.0354374.r002

23 Jun 2026

Dear Editor and Reviewer

Thank you for your letter and for the reviewer's comments concerning our manuscript entitled “Psychometric properties of the Persian version of the Chemotherapy Side Effects Fear Scale among Iranian cancer patients ". Those comments are all valuable and very helpful for revising and improving our paper, as well as providing important guidance for our research. We have carefully studied the comments and made corrections that we hope will meet with approval. The revised portion is marked with Track Changes in the paper. The main corrections in the paper and the responses to Editor and Reviewer comments are as follow:

=====================================================================

1. Editor:

1.1 Comment: Please ensure that your manuscript meets PLOS ONE's style requirements, including those for file naming. The PLOS ONE style templates can be found at

https://journals.plos.org/plosone/s/file?id=wjVg/PLOSOne_formatting_sample_main_body.pdf and

https://journals.plos.org/plosone/s/file?id=ba62/PLOSOne_formatting_sample_title_authors_affiliations.pdf

Response:

Thank you for pointing this out. We carefully made all corrections needed.

1.2 Comment: We note that the grant information you provided in the ‘Funding Information’ and ‘Financial Disclosure’ sections do not match.

When you resubmit, please ensure that you provide the correct grant numbers for the awards you received for your study in the ‘Funding Information’ section.

Response:

Thank you for pointing this out. We carefully reviewed the information provided in the Funding Information and Financial Disclosure sections and corrected the inconsistency. Both sections have now been revised to accurately state that this study was funded by the Nursing and Midwifery Care Research Center, Tehran University of Medical Sciences, Tehran, Iran, under grant number 57727.

1.3 Comment: Thank you for stating the following financial disclosure:

“The author(s) received no specific funding for this work.”

At this time, please address the following queries:

a) Please clarify the sources of funding (financial or material support) for your study. List the grants or organizations that supported your study, including funding received from your institution.

b) State what role the funders took in the study. If the funders had no role in your study, please state: “The funders had no role in study design, data collection and analysis, decision to publish, or preparation of the manuscript.”

c) If any authors received a salary from any of your funders, please state which authors and which funders.

d) If you did not receive any funding for this study, please state: “The authors received no specific funding for this work.”

Please include your amended statements within your cover letter; we will change the online submission form on your behalf.

Response:

Thank you for this clarification. We confirm that this study was funded by the Nursing and Midwifery Care Research Center, Tehran University of Medical Sciences, Tehran, Iran, under grant number 57727. The funders had no role in study design, data collection and analysis, decision to publish, or preparation of the manuscript. No authors received a salary from the funder for this study. We have included the amended financial disclosure statement in the cover letter as requested.

1.4 Comment: Your ethics statement should only appear in the Methods section of your manuscript. If your ethics statement is written in any section besides the Methods, please delete it from any other section.

Response:

Thank you for this comment. We have revised the manuscript accordingly. The ethics statement now appears only in the Methods section under “Ethical considerations.” The duplicate ethics approval and consent statement that appeared in the Declaration section has been removed from the revised manuscript.

1.5 Comment: Thank you for providing your underlying data as Supporting Information. We note that the data set contains text or data that is not in English. Please note that PLOS is an English-language publisher, so we require data sets to be provided in English as well. Please upload an English-language version of your data set.

This will also allow us to determine if your data follows PLOS standards per our Data Availability policy here: https://journals.plos.org/plosone/s/data-availability.

Response:

We have uploaded an English-language version of the de-identified dataset as Supporting Information file S1 Dataset. All variable names, value labels, category labels, and explanatory text have been translated into English.

1.6 Comment: Please include captions for your Supporting Information files at the end of your manuscript, and update any in-text citations to match accordingly. Please see our Supporting Information guidelines for more information: http://journals.plos.org/plosone/s/supporting-information.

Response:

Thank you for this comment. We have added a Supporting Information caption at the end of the manuscript and updated the in-text citation and Data Availability statement accordingly. The English-language de-identified dataset is cited in the manuscript as “S1 Dataset,” and the caption has been added at the end of the manuscript as: “S1 Dataset. De-identified minimal dataset underlying the findings of the study.”

1.7 Comment: If the reviewer comments include a recommendation to cite specific previously published works, please review and evaluate these publications to determine whether they are relevant and should be cited. There is no requirement to cite these works unless the editor has indicated otherwise.

Response:

Thank you for this clarification. We carefully reviewed and evaluated the relevant previously published literature in relation to the aims, methodology, and discussion of the present study. Based on this evaluation, the reference list has been updated where scientifically appropriate. We did not add references solely on the basis of recommendation; only publications that were considered relevant to the manuscript were included.

=====================================================================

Reviewer 1:

1.1 Comment: The abstract does not reference the specific model used for the translation of the instrument or define the key psychometric performance indicators employed in the evaluation.

Response: We sincerely thank the reviewer for this valuable comment. As suggested, we have revised the abstract to clearly specify the translation model and the key psychometric performance indicators employed in the evaluation. The translation process is now explicitly described as a forward–backward translation and cross-cultural adaptation based on the Beaton et al. guidelines. Additionally, we clarified that the psychometric evaluation was conducted according to COSMIN standards and included assessments of face validity, content validity (CVR and CVI), construct validity through exploratory and confirmatory factor analyses, convergent and discriminant validity, internal consistency (Cronbach’s alpha and McDonald’s ω), and test–retest reliability (ICC).

1.2 Comment: The source cited for cancer prevalence data appears outdated and should be updated. The authors disproportionately emphasize assessment tools for stress, depression, and anxiety over fear. Consequently, the rationale and necessity for the cross-cultural adaptation of a fear-specific assessment tool for the Iranian context are insufficiently articulated.

Response:

Thank you for your valuable comment. The information related to cancer prevalence has been updated using the most recent available data, and the revised sections have been highlighted in the manuscript for clarity. Also, we have revised the Introduction to clarify the conceptual distinction between fear and general psychological distress and to emphasize that fear of chemotherapy side effects is a treatment-specific construct associated with clinically relevant outcomes. Accordingly, we have strengthened the rationale for the cross-cultural adaptation and validation of a fear-specific instrument for the Iranian context.

1.3 Comment: The methodology section omits a reference to the specific translation model adopted (e.g., the World Health Organization guidelines or another established framework) and does not detail its distinct phases. Only five generic steps are listed without citing a credible, verifiable source to substantiate this approach. Furthermore, the type of Content Validity Ratio (CVR) calculation applied (i.e., whether relaxed or strict criteria were used) and the method for calculating the Scale Content Validity Index (SCVI) are not clearly specified. The procedures for measuring the agreement coefficient (Kappa) and conducting item analysis before factor analysis are also not described.

Response:

Thank you for this important comment. The Methodology section has been revised to explicitly state that the translation and cross-cultural adaptation process was conducted in accordance with the guidelines proposed by Beaton et al. The distinct phases of the model (forward translation, synthesis, back translation, expert committee review, pre-testing, and documentation) are now clearly described, and the appropriate reference has been added to substantiate the adopted framework. The Methodology section has been revised to explicitly describe the CVR calculation based on Lawshe’s strict criterion, the computation of the Item-level Content Validity Index (I-CVI), and the procedures for item analysis before exploratory factor analysis, including corrected item–total correlations and distributional indices. These additions clarify the statistical procedures applied in the validation process.

1.4 Comment: The findings provide an inadequate account of the modification or revision of terms, items, and cultural adaptations during the assessment of face validity, content validity, and item analysis. Additionally, the procedure for administering the preliminary test to establish sample adequacy for confirmatory factor analysis is not reported. The calculations for the Minimal Important Change (MIC), Minimal Detectable Change (MDC), and the tests for ceiling/floor effects and response ease are not clearly delineated. The presentation of the final, adapted instrument and a clear scoring guide is also lacking.

Response:

Thank you for this valuable comment. We revised the manuscript to provide a clearer description of the cross-cultural adaptation process, face validity, content validity, preliminary item analysis, CFA sample adequacy, measurement error, and scoring procedure.

We added details on the expert committee review, cognitive interviews, cultural appropriateness, response ease, and minor wording revisions made to improve clarity and naturalness of the Persian version. We clarified that no item was added or removed during the translation, face validity, or content validity stages.

We also added a new subsection on preliminary item analysis and measurement error. Corrected item–total correlations ranged from 0.383 to 0.783, all exceeding the 0.30 criterion. Missing or incomplete responses were minimal. No substantial floor or ceiling effects were observed, as all values were below the predefined 15% threshold.

Measurement error was also reported. Based on the test–retest data, SEM was 1.64 and MDC95 was 4.56.

Measurement error was also reported. Based on the test–retest data, the SEM was 1.64 and the MDC95 was 4.56. Because the study did not include a longitudinal external anchor or patient-rated global change criterion, an anchor-based MIC was not estimated. However, to provide supportive evidence for score interpretability, a distribution-based MIC estimate was calculated using the small effect size criterion. The distribution-based MIC estimate for the total CheSeFS score was 1.01 points.

We further clarified that CFA was conducted on an independent sample of 300 participants. Considering the final 11-item structure, this provided more than 25 participants per retained item, supporting sample adequacy for CFA.

Finally, we added a section presenting the final adapted 11-item Persian CheSeFS and its scoring guide. The final instrument and scoring instructions have also been provided as Supporting Information file S2 File.

1.5 Comment: While the discussion is consistent with the reported findings, addressing the deficiencies identified in the methodology and results would necessitate a corresponding enrichment of the discussion section.

Response:

Thank you for this helpful comment. We enriched the Discussion section to reflect the methodological and results-related revisions. Specifically, we added discussion of cognitive debriefing, cultural appropriateness, response ease, item homogeneity, floor and ceiling effects, measurement error indices including SEM and MDC95, the supportive distribution-based MIC estimate, and the implications of the final 11-item adapted scale and scoring guide. We also clarified the interpretation of score change by noting that the distribution-based MIC estimate should not be interpreted as an anchor-based or patient-perceived MIC, because longitudinal change data, an external anchor, and patient-rated global change criteria were not collected. The Limitations section was also revised to state that responsiveness and anchor-based MIC should be examined in future longitudinal studies.

=====================================================================

Reviewer 2:

2.1 Comment: The manuscript repeatedly uses the term “cancer patients”, which reflects a labeling-based approach. Accordingly, the authors are encouraged to replace this terminology with “patients with cancer” throughout the manuscript. This revision would improve the ethical tone of the paper and align it with internationally accepted standards for respectful and person-centered scientific communication.

Response:

Thank you for your thorough feedback. All requested revisions have been implemented.

2.2 Comment: The study is cross-sectional, yet predictors are discussed in quasi-causal language.

Response:

Thank you for this important comment. We agree that, because of the cross-sectional design of the study, the regression findings should be interpreted as associations rather than causal relationships. We revised the Discussion and Conclusion sections accordingly. Specifically, we replaced quasi-causal terms such as “predictors,” “shaping,” “leading to,” “amplifying,” and “reducing” with more cautious language such as “variables associated with,” “may be associated with,” and “linked to.” We also added a clear statement that the identified demographic and clinical variables should not be interpreted as causes of chemotherapy-related fear. In addition, we noted that future longitudinal studies are needed to clarify temporal and causal relationships.

2.3 Comment: The authors claim that the instrument was evaluated based on COSMIN criteria; however, key COSMIN domains—particularly measurement error—were not assessed or reported. If COSMIN is cited as the guiding framework, all relevant domains should be systematically evaluated and documented, or the claim should be revised accordingly.

Response:

Thank you for this important comment. We revised the manuscript to avoid implying that all COSMIN domains were fully evaluated. The text now states that the selection and reporting of measurement properties were guided by relevant COSMIN recommendations.

We also added measurement error analysis to the Methods and Results sections, including the standard error of measurement (SEM) and the minimal detectable change at the 95% confidence level (MDC95). The SEM and MDC95 for the total CheSeFS score were 1.64 and 4.56, respectively. In addition, to provide supportive evidence for score interpretability, we calculated a distribution-based MIC estimate using the small effect size criterion, resulting in an estimated MIC of 1.01 points for the total score.

We further clarified that an anchor-based MIC, responsiveness, and criterion validity were not evaluated because the study did not include longitudinal change data, an external anchor, patient-rated global change criteria, or an appropriate gold standard. This limitation has also been addressed in the Discussion and Limitations sections.

Attachment Submitted filename: Response to reviewers 27.03.1405.docx

# Peer review: Decision Letter 1

**DOI:** 10.1371/journal.pone.0354374.r003

**Authors:** Fatemeh Zarei

7 Jul 2026

Psychometric properties of the Persian version of the Chemotherapy Side Effects Fear Scale among Iranian Patients with Cancer

PONE-D-25-65182R1

Dear Dr. Reza Negarandeh

We’re pleased to inform you that your manuscript has been judged scientifically suitable for publication and will be formally accepted for publication once it meets all outstanding technical requirements.

Within one week, you’ll receive an e-mail detailing the required amendments. When these have been addressed, you’ll receive a formal acceptance letter and your manuscript will be scheduled for publication.

An invoice will be generated when your article is formally accepted. Please note, if your institution has a publishing partnership with PLOS and your article meets the relevant criteria, all or part of your publication costs will be covered. Please make sure your user information is up-to-date by logging into Editorial Manager at Editorial Manager® and clicking the ‘Update My Information' link at the top of the page. For questions related to billing, please contact billing support.

If your institution or institutions have a press office, please notify them about your upcoming paper to help maximize its impact. If they’ll be preparing press materials, please inform our press team as soon as possible -- no later than 48 hours after receiving the formal acceptance. Your manuscript will remain under strict press embargo until 2 pm Eastern Time on the date of publication. For more information, please contact onepress@plos.org.

Kind regards,

Fatemeh Zarei, PhD

Academic Editor

PLOS One

Additional Editor Comments (optional):

Reviewers' comments:

Reviewer's Responses to Questions

**Comments to the Author**

Reviewer #1: All comments have been addressed

**********

2. Is the manuscript technically sound, and do the data support the conclusions?

Reviewer #1: Yes

**********

3. Has the statistical analysis been performed appropriately and rigorously?

Reviewer #1: Yes

**********

4. Have the authors made all data underlying the findings in their manuscript fully available?

The PLOS Data policy

Reviewer #1: Yes

**********

5. Is the manuscript presented in an intelligible fashion and written in standard English?

Reviewer #1: Yes

**********

Reviewer #1: Thank you for your efforts Seems that all comments were considered.

**********

what does this mean?). If published, this will include your full peer review and any attached files.

If you choose “no”, your identity will remain anonymous but your review may still be made public.

**Do you want your identity to be public for this peer review?**  For information about this choice, including consent withdrawal, please see our Privacy Policy

Reviewer #1: **Yes:** Fazlollah Ahmadi

**********

# Editor report: Acceptance letter

**DOI:** 10.1371/journal.pone.0354374.r004

**Authors:** Fatemeh Zarei

PONE-D-25-65182R1

PLOS One

Dear Dr. Negarandeh,

I'm pleased to inform you that your manuscript has been deemed suitable for publication in PLOS One. Congratulations! Your manuscript is now being handed over to our production team.

At this stage, our production department will prepare your paper for publication. This includes ensuring the following:

* All references, tables, and figures are properly cited

* All relevant supporting information is included in the manuscript submission,

* There are no issues that prevent the paper from being properly typeset

You will receive further instructions from the production team, including instructions on how to review your proof when it is ready. Please keep in mind that we are working through a large volume of accepted articles, so please give us a few days to review your paper and let you know the next and final steps.

Lastly, if your institution or institutions have a press office, please let them know about your upcoming paper now to help maximize its impact. If they'll be preparing press materials, please inform our press team within the next 48 hours. Your manuscript will remain under strict press embargo until 2 pm Eastern Time on the date of publication. For more information, please contact onepress@plos.org.

You will receive an invoice from PLOS for your publication fee after your manuscript has reached the completed accept phase. If you receive an email requesting payment before acceptance or for any other service, this may be a phishing scheme. Learn how to identify phishing emails and protect your accounts at https://explore.plos.org/phishing.

If we can help with anything else, please email us at customercare@plos.org.

Thank you for submitting your work to PLOS ONE and supporting open access.

Kind regards,

PLOS ONE Editorial Office Staff

on behalf of

Dr. Fatemeh Zarei

Academic Editor

PLOS One
